# Arts engagement, active inference, and allostatic regulation: a neurobiological framework for understanding the health effects of aesthetic experience

**DOI:** 10.3389/fncom.2026.1852750

**Published:** 2026-08-11

**Authors:** Pier Luigi Sacco

**Affiliations:** 1 Department of Neuroscience, Imaging and Clinical Sciences, University “G. d’Annunzio” of Chieti-Pescara, Chieti, Italy; 2 metaLAB (at) Harvard, Cambridge, MA, United States

**Keywords:** active inference, allostasis, allostatic load, arts and health, interoception, metabolomics, neuroaesthetics, predictive processing

## Abstract

A growing body of evidence indicates that arts engagement produces measurable effects on mental and physical health, yet a mechanistic account linking aesthetic experience to physiological regulation remains lacking. This review proposes a neurobiological framework grounded in two convergent theoretical traditions: the active inference formulation of brain function and the allostatic model of physiological regulation. Both frameworks describe organisms as anticipatory systems that maintain viability through model-based predictive control. When active inference chronically fails as in sustained stress, trauma, or learned helplessness, the resulting allostatic load produces systemic metabolic dysregulation and disease vulnerability. This review argues that arts engagement provides contexts optimally suited to restoring effective active inference across sensorimotor, cognitive, affective, and social timescales, thereby recalibrating allostatic regulation. Arts engagement is positioned not as the only activity capable of producing such effects, but as a distinctive and potentially major instance of structured agentic experience, distinguished by its simultaneous engagement of multiple levels of the predictive hierarchy, its intrinsic epistemic motivation, and its capacity to sustain graded uncertainty without pragmatic consequence. A schematic computational specification of the framework is provided, identifying the generative model structure, precision parameters, and expected free energy decomposition relevant to arts-mediated allostatic recalibration. The framework is systematically compared with six alternative accounts of arts-health effects, showing where predictions converge and diverge. Developmental and lifespan perspectives are elaborated, with specific predictions for critical-period effects. The framework generates directional hypotheses for metabolomic, neuroendocrine, and neuroimaging studies, including predictions that distinguish the proposed mechanism from generic stress reduction.

## 1 Introduction

The relationship between arts engagement and health outcomes has moved, over the past two decades, from anecdotal observation to systematic empirical inquiry. Large-scale epidemiological studies have documented associations between cultural participation and reduced all-cause mortality, lower incidence of depression, improved cognitive function in aging, and enhanced immune markers (Fancourt and Steptoe, 2019; Stuckey and Nobel, 2010). Randomized controlled trials have shown that specific arts interventions produce measurable effects on cortisol levels, inflammatory biomarkers, heart rate variability, and subjective wellbeing (Fancourt et al., 2016; Dunbar et al., 2012). The World Health Organization’s scoping review of over 3,000 studies concluded that arts engagement constitutes a significant, independent determinant of health (Fancourt and Finn, 2019). Systematic reviews across specific domains such as music (MacDonald et al., 2012), visual arts (Camic, 2008), dance (Koch et al., 2014), and multi-modal interventions (Sheppard and Broughton, 2020) converge on this finding across diverse populations and outcome measures. Longitudinal evidence now links sustained arts engagement to flourishing in young adults (Bone et al., 2023). A comprehensive taxonomy has recently identified at least fifty distinct mechanisms through which arts engagement may influence mental health (Fancourt et al., 2026).

This evidence base is not uncontested, and acknowledging its limitations strengthens the case for the present undertaking. The WHO scoping review in particular has been criticized for aggregating studies of uneven methodological quality without appraising that quality, so that its strong conclusions outrun the rigor of the underlying evidence (Clift, 2020). More pointedly, Skov and Nadal (2025) have argued that the studies most often cited as demonstrating health benefits of the arts lack adequate clinical-trial procedures and statistical analysis, and, crucially, that they rarely specify the physiological processes through which art is supposed to affect the etiology of disease, concluding that there is as yet no strong evidence that arts-based interventions improve health. These arguments have themselves been contested: in a series of commentaries, researchers and practitioners in the field have argued that the critique holds arts-based interventions to standards not applied to other accepted treatments, that its demand for an art-specific mechanism misconstrues how psychotherapy and many pharmacological agents work, since these operate through common or distributed mechanisms rather than a single dedicated pathway, that it conflates distinct practices ranging from self-directed arts engagement to formally regulated arts therapies, and that its neuroscientific premises sit uneasily with a network neuroscience in which functional effects are emergent properties of distributed network reconfiguration rather than outputs of localized, stimulus-specific modules (Karkou et al., 2025; Vessel, 2025). The debate thus remains open, and the present review does not attempt to settle it; what the opposing positions share is the recognition that the field would be advanced by an explicit account of the processes through which aesthetic experience could influence physiological state, and the appropriate response to the controversy is to supply such an account rather than to abandon the field.

A literature that is mechanistically underspecified and methodologically heterogeneous is precisely the kind of literature that stands to gain from an explicit, falsifiable account of how aesthetic experience could produce physiological change, an account that names the processes involved and the measurements that would confirm or refute their operation. The framework developed here is offered in that spirit. It specifies a candidate computational mechanism, as the critics ask, yet one that is explicitly not art-specific, since arts engagement is treated as a distinctive configuration of general predictive-processing capacities rather than a dedicated, art-unique pathway, in keeping with the respondents’ argument that a therapeutic mechanism need not be unique to the arts to be genuine. Its value lies less in adding another reported association than in specifying the causal architecture that would render existing findings interpretable and would discipline the design of the better-controlled studies that all sides agree are needed.

Yet the field confronts a persistent theoretical gap. Most accounts remain fundamentally descriptive: they document correlations, enumerate mediators, and invoke broad psychological constructs like stress reduction, social bonding, emotional regulation, or self-efficacy, without specifying the computational architecture through which aesthetic experience produces physiological change. The result is a rapidly expanding catalog of effects without a unifying explanatory framework. Fancourt et al.’s (2026) taxonomy, for instance, identifies 50 mechanisms spanning psychological, neurological, biological, and social processes, and explicitly concludes that no single mechanism predominates; rather, a diverse network of interconnected processes operates in synergy. The present review proposes that active inference provides the computational architecture that may integrate this mechanistic diversity: the 50 mechanisms can be understood as manifestations, at different hierarchical levels and timescales, of the same underlying process of predictive model updating and precision recalibration. Why should interpreting a poem, improvising a melody, or coordinating a group dance reduce inflammatory markers or recalibrate cortisol rhythms? What is the mechanism bridging the gap between the psychological experience of artistic engagement and its downstream metabolic consequences?

This review proposes that the missing link is provided by the convergence of two theoretical frameworks that share a deep computational logic: the active inference formulation of brain function (Friston, 2010; Parr et al., 2022) and the allostatic model of physiological regulation (Sterling, 2012; McEwen, 1998; Schulkin and Sterling, 2019). Both describe organisms as fundamentally predictive systems that maintain viability not by reacting to perturbations but by anticipating them. Both locate pathology in the chronic failure of anticipatory control. And both converge on the insight that activities providing graded opportunities for successful prediction, action, and outcome confirmation can restore allostatic regulation, with arts engagement representing a distinctive instance of such activity.

A note on scope is needed at the outset. The framework developed here does not claim that arts engagement is the only activity capable of restoring active inference and recalibrating allostatic regulation. Sports, games, skilled crafts, and other forms of structured agentic experience share many of the relevant computational features. It should also be acknowledged that the category “art” is itself contested; Skov and Nadal (2020) have argued that “art” does not constitute a natural kind for psychological or neuroscientific purposes, and that aesthetic processes operate identically whether their objects are classified as art or not. The present framework is compatible with this position: the claim is not that art possesses a unique neural substrate, but that activities conventionally grouped under “arts engagement” tend to combine certain computational features in a configuration that is distinctive, though not exclusive, and that thereby occupies a distinctive position within the broader landscape of beneficial activities. Whether these activities combine the relevant features more reliably than others is an empirical question, addressed in Section 7, where the conditions under which non-arts activities would be predicted to produce equivalent effects are also specified. From the perspective of predictive coding, artistic culture functions as a “playground for the predictive brain” (Bortolotti et al., 2024), offering flexible simulation spaces in which generative models can be tested and updated without the constraints of pragmatic consequence. This claim is empirically testable and potentially falsifiable, and the conditions for its falsification are specified below.

A note on the nature of this review is also in order. This is a narrative, theoretical review rather than a systematic one: its aim is conceptual integration across four literatures that have developed largely in parallel, namely the active inference and free energy formulation of brain function, the allostatic model of physiological regulation, neuroaesthetics and the predictive processing of art, and the epidemiology of arts and health. Because the goal is the construction and evaluation of a mechanistic framework rather than the exhaustive quantification of a defined effect, the literature was selected to establish the theoretical foundations of each contributing tradition and to identify the most directly relevant recent empirical and conceptual work, with priority given to foundational sources and to studies that bear on the specific computational claims developed here. No systematic search protocol or formal inclusion criteria were applied, and the review accordingly makes no claim to a comprehensive coverage of the arts-and-health literature; its contribution is integrative and hypothesis-generating rather than evidential synthesis.

## 2 Predictive processing and active inference

The predictive processing framework represents a fundamental reconceptualization of neural computation. Instead of treating the brain as a primarily reactive system that processes incoming sensory signals bottom-up, this framework proposes that cortical computation is dominated by top-down predictions about the causes of sensory inputs (Rao and Ballard, 1999; Friston, 2005; Clark, 2013). The brain generates a hierarchical generative model: structured expectations about what sensory signals should look like given its current model of the environment, and continuously compares these predictions against incoming data. The residual prediction error propagates upward through the cortical hierarchy, driving model updating only insofar as current predictions fail to account for sensory evidence. Perception, on this account, is an active construction: the brain’s best inference about the hidden causes generating its sensory data (Hohwy, 2013).

A critical component of this architecture is precision weighting, i.e., the confidence the system assigns to its own expectations versus incoming data. Precision determines how much prediction errors drive model updating: high-precision errors command revision; low-precision ones are attenuated (Feldman and Friston, 2010). Computationally, precision is thought to be implemented through the gain modulation of neural populations encoding prediction errors. On the active inference account, this gain modulation is regulated by neuromodulatory systems: dopamine is interpreted as encoding precision over reward and policy prediction errors, acetylcholine as modulating sensory precision, norepinephrine as signaling unexpected uncertainty, and serotonin as modulating the precision of prior beliefs and the temporal horizon over which predictions are evaluated (Friston et al., 2014; Carhart-Harris and Friston, 2019). Serotonin’s role in precision weighting is less well characterized than that of the other three systems, but converging evidence from computational modeling and pharmacological studies suggests that serotonergic signaling regulates the balance between prior expectations and incoming evidence, with implications for mood, patience, and the temporal discounting of future outcomes. This is relevant to the present framework because chronic allostatic dysregulation degrades serotonergic function through the inflammatory-kynurenine pathway (Section 10), potentially disrupting the precision of prior beliefs and contributing to the characteristic hopelessness and foreshortened temporal perspective of depression.

While the specific mapping from neuromodulators to precision remains an active area of investigation, and alternative interpretations exist, the broad convergence between neuromodulatory function and precision control is increasingly supported (Parr et al., 2022). It should be noted that this mapping is a useful heuristic rather than a strict one-to-one correspondence: dopamine, for instance, plays roles in motor control, working memory, and salience that extend beyond reward precision, and each neuromodulatory system participates in multiple computational functions. The claims that follow about neuromodulatory targets of arts-mediated recalibration should be understood as identifying the primary precision-related functions of these systems, not as implying that each transmitter serves only a single computational role. This neuromodulatory connection is crucial for the present argument, because these same transmitter systems are primary targets of allostatic dysregulation under chronic stress, providing a direct computational bridge between neuromodulatory disturbance and the failure of predictive control that, on the present account, underlies allostatic pathology.

Active inference extends this logic from perception to action (Friston, 2010; Friston et al., 2017). Organisms do not merely update models to match the world; they act on the world to make it match their predictions. Crucially, organisms select actions by evaluating expected free energy, a quantity that decomposes into pragmatic value (the expected achievement of preferred outcomes) and epistemic value (the expected reduction of uncertainty about the world’s hidden states; Friston et al., 2017; Schwartenbeck et al., 2019). This decomposition has implications developed in Section 6: certain activities may be distinctive in the degree to which epistemic value (the intrinsic drive to explore, resolve ambiguity, and refine models) dominates over pragmatic value. The felt reward of resolving an ambiguity or mastering a pattern signals successful model updating, not the achievement of any external goal. This epistemic motivation corresponds at the neural level to the activation of dopaminergic circuits encoding anticipatory reward linked to prediction and uncertainty resolution (Schwartenbeck et al., 2019), connecting the phenomenology of curiosity-driven exploration to the neurocomputational mechanisms of uncertainty reduction. The specific relevance of this mechanism to aesthetic experience is suggested by Salimpoor et al’s (2011) demonstration that intense musical pleasure is accompanied by striatal dopamine release, with the caudate more involved during the anticipation of peak emotional responses and the nucleus accumbens during their experience, though the anticipatory component is best characterized as evidence for anticipatory reward signaling rather than for epistemic value in the technical active inference sense.

This framework has received substantial empirical support from neuroimaging studies reporting neural functioning compatible with predictive coding in the visual cortex (Alink et al., 2010), auditory cortex (Heilbron and Chait, 2018), and somatosensory processing (Adams et al., 2013), as well as from computational modeling of decision-making under uncertainty (Schwartenbeck et al., 2015), interoceptive inference (Seth and Friston, 2016), and sensorimotor control (Wang et al., 2014). The key insight for the present argument is that wellbeing is tied to the capacity for effective active inference: the ability to build accurate generative models, generate reliable predictions, act to bring about predicted states, flexibly update when predictions fail, and appropriately calibrate the precision assigned to prediction errors at each level of the hierarchy.

## 3 When active inference fails: the computational basis of stress pathology

If wellbeing depends on effective active inference, then psychopathology can be understood as its systematic breakdown, a reframing extensively developed in computational psychiatry (Stephan et al., 2016; Smith et al., 2021; Paulus et al., 2019). This perspective offers a unified computational account of conditions typically treated as nosologically distinct.

Depression, in this framework, corresponds to a state in which action-outcome contingencies have collapsed. The organism’s generative model has learned that no available action reliably reduces prediction error, a computational formalization of learned helplessness (Huys and Dayan, 2009; Barrett et al., 2016). The precision assigned to action-dependent prediction errors is chronically low: the system has effectively concluded that action is uninformative. On Barrett’s (2017) constructionist account, the brain’s failure to generate accurate interoceptive predictions in this state produces not merely “sad mood” but a wholesale disruption of the affective inferences that organize bodily regulation, social cognition, and goal-directed behavior. The metabolic consequences are direct: the brain’s interoceptive model, unable to generate reliable predictions about the body’s energy needs, defaults to a conservative, high-inflammation, low-energy configuration corresponding clinically to the somatic symptoms of depression: fatigue, sleep disturbance, appetite change, psychomotor retardation.

Anxiety, by contrast, reflects excessive uncertainty about the effects of action. The generative model assigns high precision to prediction errors but cannot resolve them: the organism anticipates threats that it cannot predict with sufficient accuracy to mount effective responses (Paulus and Stein, 2006; Hein et al., 2021). Within the precision framework, the noradrenergic system, interpreted as signaling unexpected uncertainty and modulating sensory gain, is chronically upregulated, producing hypervigilance and a sustained anticipatory state that fails to resolve. Trauma introduces a more radical disruption: generative models encoding unpredictable, uncontrollable threats shatter the basic assumption that the organism can exert causal influence on its environment (Linson and Friston, 2019). The sense of agency is fundamentally compromised, producing the characteristic oscillation between re-experiencing (intrusive prediction error signals that overwhelm the model) and avoidance (active suppression of model updating in threat-relevant domains).

Chronic stress, even without meeting diagnostic criteria for any specific disorder, produces a subtler but pervasive degradation of active inference capacity. When the environment persistently generates prediction errors that cannot be resolved through available actions, as in conditions of social adversity, economic precarity, or relational instability, the system maintains costly anticipatory states without resolution. The organism thus remains in chronic prediction error: expecting threats, preparing responses, mobilizing physiological resources, but without the successful action completion that would allow these anticipatory states to discharge. This unresolved anticipatory activation has direct physiological consequences, because the same predictive architecture that governs perception and action also governs physiological regulation.

Importantly, chronic stress degrades precision weighting at multiple levels of the hierarchy simultaneously. At the sensory level, sustained noradrenergic upregulation produces attentional hypervigilance, that is, excessive precision on threat-relevant exteroceptive signals at the expense of flexible context-sensitivity. At the level of action selection, repeated failure erodes the precision assigned to action-dependent prediction errors, producing the motivational withdrawal characteristic of learned helplessness. At the social level, chronic adversity degrades the precision of interpersonal predictions, leading to social hypervigilance or withdrawal, a reduced capacity to model others’ intentions accurately, which further erodes the social co-regulation that normally buffers allostatic load. This multi-level precision degradation is significant because it implies that effective interventions must address precision calibration across hierarchical levels, a point that motivates the domain-specific analysis in Section 8 and the art-form-specific predictions in Section 10.

## 4 Allostasis as physiological active inference

The concept of allostasis (Sterling and Eyer, 1988; McEwen, 1998; Sterling, 2012; Schulkin and Sterling, 2019) describes physiological regulation as the anticipatory adjustment of parameters in advance of predicted demands. The brain predicts upcoming metabolic needs based on contextual cues (time of day, learned associations, social signals, environmental threats) and preemptively adjusts hormonal, cardiovascular, immune, and metabolic parameters to meet anticipated requirements. A note of conceptual history is important: Sterling and McEwen, though both contributing to allostatic theory, held significant disagreements (Ramsay and Woods, 2014). Sterling emphasized anticipatory optimization and explicitly framed allostasis as a predictive, brain-centered mode of regulation, criticizing McEwen’s allostatic load concept as insufficiently distinct from homeostatic thinking. McEwen focused on the cumulative physiological cost of sustained allostatic activation. The active inference interpretation developed here aligns more naturally with Sterling’s formulation, while drawing on McEwen’s operationalization of allostatic load for empirical predictions.

The structural correspondence between allostasis and active inference is deep (Stephan et al., 2016; Seth and Friston, 2016; Petzschner et al., 2021). Both describe anticipatory, model-based regulation that minimizes prediction errors through proactive control. However, claiming a formal computational identity would require specifying the generative model, prediction errors, and precision parameters in allostatic terms with mathematical rigor, a project that remains incomplete, though Stephan et al.’s allostatic self-efficacy model and Barrett et al.’s (2016) active inference account of interoception in depression represent important steps. What can be stated is that allostatic regulation is predictive rather than reactive, minimizes discrepancies between expected and actual physiological states, and is orchestrated by an integrated allostatic-interoceptive network spanning prefrontal, insular, and subcortical circuits (Kleckner et al., 2017; Barrett and Simmons, 2015).

Interoception specifically plays a central role. On the predictive account, the anterior insula computes prediction errors for visceral signals, comparing top-down interoceptive predictions against bottom-up viscerosensory inputs (Craig, 2009; Seth, 2013; Seth and Friston, 2016). Barrett’s (2017) constructionist theory extends this by proposing that the brain constructs emotional experience from interoceptive predictions: affect is the brain’s running commentary on the body’s predicted metabolic state. It should be noted that the constructionist framework remains debated; basic emotion theorists argue for dedicated neural circuitries for discrete emotional categories. The present argument does not require the full constructionist ontology but draws on the specific, more widely shared claim that interoceptive prediction accuracy modulates the efficiency of allostatic regulation. The vagus nerve, as the primary conduit for afferent visceral information to the brain, is a key anatomical substrate for this process; vagal tone, indexed by respiratory sinus arrhythmia, is an established marker of allostatic flexibility (Thayer et al., 2012) and is directly modulated by singing, rhythmic breathing, and other practices integral to many arts forms (Bernardi et al., 2001; Vickhoff et al., 2013). Owens et al. (2018) have further elaborated the computational framework for interoceptive inference, showing how precision-weighted interoceptive prediction errors can account for individual differences in emotional awareness and autonomic regulation, a perspective that directly supports the interoceptive mechanism developed in Section 9. Inaccurate interoceptive models produce inefficient allostatic regulation: the system mobilizes resources it does not need, or fails to mobilize resources it needs, generating the metabolic waste that accumulates as allostatic load.

## 5 From failed active inference to allostatic load: the pathway to disease

Proceeding on the basis of this structural correspondence, even in advance of a formal proof of computational identity, the pathway from psychological stress to physical disease becomes tractable. When active inference fails at the psychological level, that is, when the organism cannot build accurate models of threats, cannot identify effective actions, or cannot confirm that actions have produced expected outcomes, the consequence is sustained anticipatory physiological activation: HPA axis engagement, sympathetic tone, immune priming, metabolic mobilization. This is not a “stress response” in the classical sense of a reaction to an acute stressor; it is the failure of the predictive system to stand down after mobilization, because the prediction error that triggered mobilization was never resolved through successful action.

Allostatic load (McEwen, 1998; McEwen and Stellar, 1993) captures the cumulative physiological cost of this unresolved anticipatory state. Operationally, it is measured as a composite index with specific biomarker components: neuroendocrine (cortisol and DHEA-S, noting that DHEA-S levels have a strong age-dependent trajectory and that age-correction is essential in cross-sectional comparisons), cardiovascular (systolic and diastolic blood pressure), metabolic (HDL/LDL cholesterol, glycosylated hemoglobin, waist-hip ratio), and immune (CRP, IL-6, fibrinogen), with defined scoring algorithms (Seeman et al., 1997, 2001; Juster et al., 2010). The relationship between chronic stress and these biomarkers is not straightforwardly linear: for instance, chronic stress can produce either hypercortisolism or hypocortisolism depending on duration, type, and developmental timing of exposure (Miller et al., 2007; Heim et al., 2000), and the trajectory from initial hyperactivation to eventual blunting follows a non-linear pattern that the framework must accommodate.

Importantly, allostatic load results not from “too much stress” but from the specific computational failure of unresolved anticipation-action-resolution cycles. An organism encountering frequent but predictable, controllable challenges (an athlete in training, a musician rehearsing a difficult passage) experiences high metabolic demand but achieves allostatic resolution through successful active inference. Consider a musician preparing for a concert: cortisol rises during rehearsal, sympathetic tone increases, and glucose is mobilized, but when the passage is mastered and the performance executed, these systems stand down. The cycle completes: resources are mobilized, deployed, and returned to baseline. Contrast this with an employee facing unpredictable evaluation from an inconsistent supervisor: cortisol rises, resources mobilize, but the uncertainty is never resolved because the supervisor’s criteria are opaque and shifting. No action reliably produces the predicted outcome. It is when this cycle is chronically interrupted, i.e., when predictions never confirm, actions never resolve, and outcomes never arrive that allostatic load accumulates. This distinction identifies with computational precision the kind of intervention that should reverse allostatic dysregulation: not the mere reduction of arousal, not the avoidance of challenge, but the restoration of effective predict-act-confirm contingencies that allow allostatic systems to mobilize, deploy, and resolve in adaptive cycles.

## 6 Toward a computational specification: a generative model for arts-mediated allostatic recalibration

A central objection to active inference accounts, and one that applies with particular force to extensions beyond established domains like sensorimotor control, is that verbal descriptions of “precision,” “generative models,” and “prediction errors” can become so flexible as to accommodate any observation (Colombo and Wright, 2017; Bowers and Davis, 2012). To mitigate this concern, this section provides a schematic computational specification of the framework, identifying the key components that would need to be formalized for quantitative modeling. This specification falls short of a full mathematical formulation, which requires an empirical parameterization not yet available, but it identifies the generative model structure, the relevant precision parameters, and the expected free energy decomposition in sufficient detail to orientate future computational work. The translation of this schematic into a fully parameterized generative model, expressed in standard variational or message-passing notation, remains a priority for future research and will require an empirical estimation of the relevant parameters from arts intervention data.

It is useful to state at the outset the epistemic status of the components introduced below, since they differ in how far they are evidentially supported. Three tiers can be distinguished. First, established components: the existence of hierarchical predictive processing at the sensorimotor, perceptual-affective, and narrative-social levels (L1 through L3), the role of precision weighting in gating prediction errors, and the predictive character of allostatic-interoceptive regulation are each supported by the substantial empirical literatures reviewed in Sections 2 and 4. Second, theoretical interpretations: the mapping of specific neuromodulators onto precision parameters and the construal of allostasis as physiological active inference are reasoned extensions of these literatures that remain debated and incompletely formalized. Third, speculative components proposed here: the allostatic prediction level (L4), the allostatic prediction error as a measurable quantity, and arts-mediated recalibration of precision parameters are novel constructs of this framework whose status is, at present, that of hypotheses awaiting test. The reader should treat L4 and the allostatic prediction error in particular as organizing proposals rather than established findings; their candidate operationalizations are set out in Section 10, and the conditions under which they would be disconfirmed are specified in the falsifiability conditions of that section.

*The hierarchical generative model*. The framework posits a hierarchical generative model with at least four levels relevant to arts-mediated allostatic regulation. At the lowest level (L1), the model generates predictions about sensorimotor states: proprioceptive signals, motor efference copies, low-level auditory and visual features. At the second level (L2), it generates predictions about perceptual objects and affective states: musical phrases, visual compositions, interoceptive states, emotional valence. At the third level (L3), it generates predictions about narrative and social contingencies: character intentions, plot trajectories, interpersonal dynamics. At the fourth level (L4), it generates predictions about allostatic demands: metabolic needs, arousal requirements, the expected physiological cost of ongoing and anticipated activities. Each level generates top-down predictions to the level below and receives bottom-up prediction errors from it. A clarification is needed regarding the relationship between L2 and L4. L2 interoceptive prediction, as characterized in standard accounts (Seth and Friston, 2016; Owens et al., 2018), concerns the moment-to-moment inference about current visceral states (e.g., heart rate, gastric motility, respiratory rhythm). L4 allostatic prediction operates at a longer timescale and higher level of abstraction: it concerns the anticipated metabolic budget for upcoming activities and the anticipated trajectory of physiological states over minutes to hours. L4 draws on L2 interoceptive signals as input but generates predictions about future allostatic demands rather than current visceral states. The neural substrates are expected to overlap partially (both involve the anterior insula and ventromedial prefrontal cortex) but to be distinguishable in their temporal dynamics: L2 prediction errors update on the timescale of seconds, whereas L4 prediction errors reflect discrepancies between anticipated and actual allostatic trajectories over longer periods. This distinction is testable using fMRI paradigms that separately manipulate momentary interoceptive surprise and longer-term metabolic expectation. L4 calls for particular caution. While L1 through L3 map onto well-documented predictive processing literatures (sensorimotor prediction, perceptual-affective inference, and social-narrative prediction, respectively), the existence of a computationally distinct level generating predictions about future metabolic demands, as opposed to a longer-timescale aspect of L2 interoceptive inference, has not been directly demonstrated. The Kleckner et al. (2017) allostatic-interoceptive network and the Barrett and Simmons (2015) framework describe interoceptive processing broadly rather than isolating a separable allostatic prediction level. The L4 construct is proposed here as a useful abstraction for organizing the framework’s predictions, and the question of whether it corresponds to a genuinely distinct computational process or to an extension of interoceptive inference along the temporal dimension is itself an empirical question that the framework identifies. Arts engagement is distinctive in simultaneously generating prediction errors at L1 through L3. The pathway from aesthetic prediction errors to allostatic modulation is hypothesized to operate through affective-interoceptive mediation rather than through direct hierarchical propagation: prediction errors at L1 through L3, to the extent that they carry affective valence (the pleasure of resolution, the tension of suspense, the reward of mastery), modulate interoceptive states at L2, which in turn update the allostatic predictions at L4. On this account, it is not the sensory or narrative prediction error per se that perturbs the allostatic system, but its affective-interoceptive consequences. This two-step pathway (aesthetic prediction error → affective-interoceptive modulation → allostatic updating) is consistent with the evidence that aesthetic experience reliably modulates autonomic states (Salimpoor et al., 2011; Lehne and Koelsch, 2015; Wassiliwizky et al., 2017) and with Barrett’s (2017) account of affect as the brain’s running commentary on allostatic state. The critical difference between arts engagement and other sources of affective-interoceptive modulation is that arts engagement generates these modulations within a context of epistemic safety and graded controllability, permitting the allostatic cycle to complete rather than accumulating as load.

*Precision parameters*. Each level of the hierarchy has associated precision parameters that determine the gain on prediction errors at that level. The framework identifies four precision parameters as particularly relevant to arts-mediated recalibration. First, π_action_ (action precision): the confidence the system places in the prediction that its actions will produce expected outcomes. This parameter is chronically low in depression (learned helplessness) and is the primary target of the prediction-action loop restoration mechanism (Section 9). Arts engagement rebuilds π_action_ by providing environments where creative actions consistently produce observable consequences. Empirically, π_action_ could be estimated using computational tasks that measure model-based versus model-free decision-making (e.g., two-step decision tasks; Daw et al., 2011), where reduced model-based control indexes low action precision. Second, *π*_intero_ (interoceptive precision): the confidence placed in interoceptive predictions about visceral states. This parameter modulates the accuracy of allostatic regulation directly. It is reduced in conditions of chronic stress and alexithymia. Embodied arts engagement (dance, theater, vocal performance) trains π_intero_ by drawing sustained attention to bodily states in contexts of safe exploration. Empirically, π_intero_ can be estimated using heartbeat detection tasks (Schandry, 1981) or heartbeat discrimination tasks (Garfinkel et al., 2015), providing a behavioral operationalization of interoceptive precision that can be measured at baseline and after intervention. Third, π_social_ (social precision): the confidence placed in predictions about others’ mental states and likely behaviors. This parameter modulates the metabolic cost of social interaction (Beckes and Coan, 2011). Collaborative and collective arts engagement trains π_social_ through joint active inference, where participants must continuously model and coordinate with co-participants’ predictions. A fourth precision parameter, π_sensory_ (sensory precision), modulates the gain on exteroceptive prediction errors and is relevant primarily to anxiety (chronically elevated) rather than to the therapeutic mechanism of arts engagement per se, though the tolerance of amplified uncertainty in aesthetic experience (Section 7) may specifically train the system’s capacity to modulate π_sensory_ flexibly. Three of these parameters attach to specific hierarchical levels (π_sensory_ to L1, π_intero_ to L2, and π_social_ to L3), whereas π_action_ is the precision on action (policy) selection and therefore operates across the hierarchy rather than residing at a single level.

*Expected free energy and aesthetic action selection*. In the active inference framework, an agent selects policies (sequences of actions) by evaluating their expected free energy G, which decomposes into pragmatic value (the expected achievement of preferred outcomes) and epistemic value (the expected reduction of uncertainty about hidden states). Formally, the expected free energy for a policy π can be written as G(π) ≈ −𝔼_Q_[*ln* *P*(*o|C*)] − 𝔼_Q_[*D*_KL_[*Q*(*s|o*, π)||*Q*(**s*|π*)]], where the first term is the (negative) pragmatic value (the expected log probability of observations o under the prior preferences C) and the second is the (negative) epistemic value (the expected information gain about hidden states s from future observations), so that the policies with the lowest expected free energy are those with the greatest combined pragmatic and epistemic value. In arts engagement, the prior preferences C are weakly specified (there is no strong preferred outcome beyond the experience itself), which reduces the pragmatic term; meanwhile, the richness and multi-level structure of aesthetic stimuli amplify the epistemic term by providing observations that are highly informative about hidden states at multiple hierarchical levels. In most everyday contexts, pragmatic value dominates: we act to eat, to earn, or to avoid danger. The framework’s claim about arts engagement can now be stated with computational precision: arts engagement is characterized by a relative dominance of the epistemic term over the pragmatic term in the expected free energy. The “goal” of interpreting a poem or improvising a melody is not pragmatic (no resource is gained, no threat avoided) but epistemic (uncertainty about the meaning of the text, the trajectory of the melody, the responses of co-participants is reduced). This epistemic dominance has a specific consequence for allostatic regulation: because the pragmatic term is minimal, the allostatic system does not prepare for the metabolic costs of real-world consequences (fight, flight, resource competition). Instead, the system mobilizes the more moderate metabolic resources associated with epistemic foraging such as attentional engagement, model updating, and reward from uncertainty resolution, and crucially, these resources are deployed and returned to baseline when the aesthetic engagement concludes, completing the allostatic cycle without generating load.

**Figure 1:**
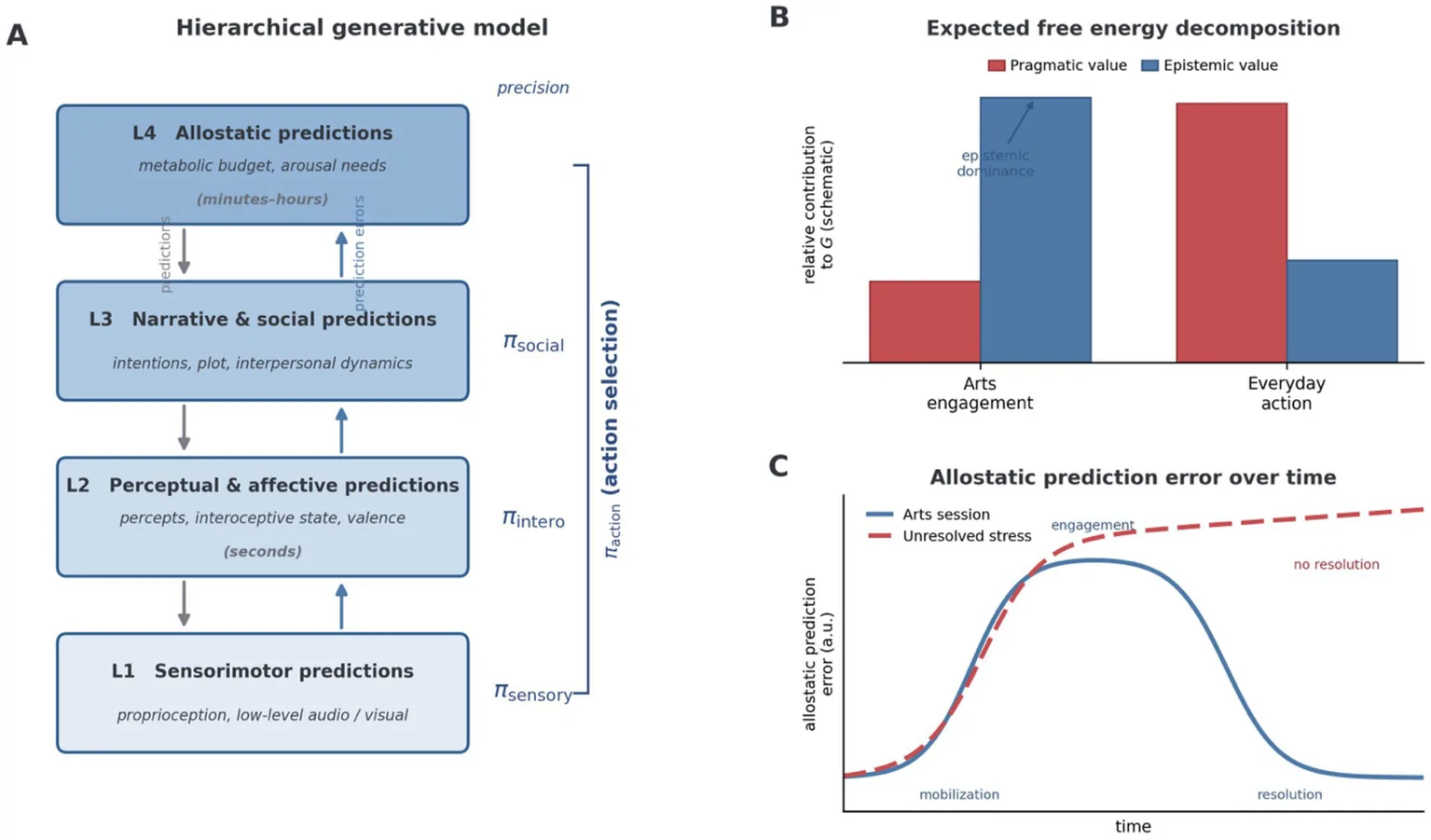
Schematic computational specification. **(A)** The four-level hierarchical generative model: L1 (sensorimotor predictions), L2 (perceptual-affective predictions), L3 (narrative-social predictions), L4 (allostatic predictions). Top-down predictions (downward arrows) and bottom-up prediction errors (upward arrows) at each level. Precision parameters π_sensory_, π_intero_, and π_social_ are indicated at their relevant levels (L1, L2, L3, respectively); π_action_, the precision on action selection, operates across the hierarchy rather than at a single level. L2 and L4 are distinguished by timescale: L2 concerns moment-to-moment visceral inference, L4 concerns anticipated allostatic trajectories. **(B)** Expected free energy decomposition for arts engagement versus everyday action: The relative dominance of the epistemic term in arts contexts. **(C)** The allostatic prediction error as a function of time during an arts session (mobilization, engagement, resolution) versus during unresolved stress (mobilization without resolution). Panels **(B,C)** are conceptual schematics that illustrate the qualitative patterns predicted by the framework; they are not derived from empirical data or numerical simulation.

*The allostatic prediction error*. The framework proposes that the critical variable linking arts engagement to health is the allostatic prediction error: the discrepancy between the brain’s prediction of the body’s metabolic state and the actual metabolic state as reported by interoceptive afferents. When this error is chronically large and unresolved, as in sustained stress, allostatic load accumulates. Arts engagement reduces allostatic prediction error through two routes: (a) by restoring the prediction-action loop (so that allostatic mobilization is followed by resolution, reducing the temporal integral of the error signal), and (b) by training interoceptive precision (so that the predictions themselves become more accurate, reducing the magnitude of the error signal at any given time point). The framework predicts that the allostatic prediction error, if it could be measured (perhaps through continuous interoceptive-autonomic mismatch indices), should decrease over the course of an effective arts intervention program, and that the rate of decrease should predict health outcomes. Figure 1 summarizes the main points.

## 7 Arts engagement as active inference training: specificity and boundary conditions

The central claim of this review is that arts engagement provides settings that are well-suited to restoring and exercising active inference capacities, and that this is the primary mechanism through which aesthetic experience produces health effects. Before developing this claim, an important qualification is necessary.

*The specificity question*. Several features of arts engagement that make it effective for active inference restoration such as clear action-outcome contingencies, controllable uncertainty, immediate feedback, or intrinsic motivation, are shared by other structured activities. Sports provide contingencies and graded challenge. Recreational video games offer precisely calibrated uncertainty gradients. Skilled crafts involve sensorimotor prediction and visible outcomes. The honest answer to “why arts?” is not that the arts uniquely possess these features, but that arts engagement combines them in a particular configuration that may make it distinctively, though not exclusively, effective. Three features distinguish arts engagement from most other structured activities.

*First, simultaneous multi-hierarchical engagement.* Arts engagement characteristically operates across multiple levels of the predictive hierarchy simultaneously: low-level sensorimotor prediction (milliseconds to seconds), mid-level perceptual and affective inference (seconds to minutes), and high-level narrative, symbolic, and social prediction (minutes to hours). A dancer simultaneously manages proprioceptive control, musical entrainment, aesthetic expression, and social coordination. A reader of fiction simultaneously processes lexical prediction, narrative anticipation, character inference, and thematic interpretation. While sports engage sensorimotor and social levels powerfully, and recreational games engage strategic levels, arts engagement is unusual in the breadth and depth of simultaneous hierarchical engagement. Bortolotti et al. (2025) have characterized experiences that simultaneously engage multiple neural networks, including the salience network, default mode network, and central executive network, in ways that resist decomposition into simpler processes as “supercomplex experiences.” Their framework, developed in the context of expert performance domains such as musical performance, visual art creation, and wine tasting, proposes that such experiences are characterized by five co-occurring criteria, including the development of specialized neural architectures through training and the emergence of irreducible gestalt properties. This characterization is compatible with the multi-hierarchical engagement claim advanced here, and its grounding in network neuroscience (specifically the coordinated activity of distributed brain systems documented through neuroimaging of expert performance) provides converging evidence from a different level of analysis. The tight coupling between perception, action, interoception, and social cognition documented in these domains (see also Vessel et al., 2012, 2013, on default mode network engagement during intense aesthetic experience; Frascaroli et al., 2024, on the broader encounter between aesthetics and predictive processing) may be what enables arts engagement to modulate allostatic regulation at L4 through coordinated prediction error processing at L1 through L3. This claim is empirically testable: if correct, neuroimaging should reveal more distributed, cross-hierarchical neural activation during arts engagement than during comparably demanding non-arts activities.

*Second, epistemic dominance over pragmatic value.* Most everyday activities, including sports and games, involve substantial pragmatic motivation: winning, scoring, completing. Arts engagement is distinctive in the degree to which epistemic value dominates, in the sense made precise by the expected free energy decomposition of Section 6: the reward of aesthetic engagement is the felt correlate of model updating itself (Van de Cruys and Wagemans, 2011; Vessel et al., 2012). This epistemic dominance may be therapeutically significant because it decouples the restoration of active inference from the very performance contingencies that may have contributed to allostatic dysregulation. Where competitive activities can themselves become sources of uncontrollable prediction error under the form of performance anxiety, fear of failure, and social comparison, the epistemic frame of aesthetic engagement provides a context in which prediction and model updating are valued for their own sake, independently of evaluative outcome. A clarification is in order here: the claim concerns the epistemic framing of the activity itself, not the participant’s overall motivational profile. A professional musician performing for income operates within a pragmatic context, but the aesthetic frame of the performance still provides moments of epistemic engagement, uncertainty resolution, and model updating that are valued for their own sake within the activity. The therapeutic mechanism is hypothesized to operate during these episodes of aesthetically framed engagement, regardless of the surrounding pragmatic context. This implies that the framework’s predictions apply most strongly to arts engagement undertaken in an epistemic frame (leisure, exploration, creative play) but are not limited to it, since even professionally motivated arts participation includes substantial episodes of epistemic engagement. Bortolotti et al. (2024) have developed this point in evolutionary terms, arguing that artistic culture provides “flexible simulation spaces” complementary to the conserving function of cultural norms: whereas norms minimize collective surprise by stabilizing social predictions, the arts enable the active exploration of novel patterns unconstrained by pragmatic risks. The epistemic dominance of aesthetic engagement can thus be understood as serving an adaptive function in maintaining the flexibility of generative models at both individual and collective levels.

*Third, tolerance of sustained and amplified uncertainty.* A critical insight from the neuroaesthetics literature is that aesthetic experience does not simply minimize prediction error; it often involves the deliberate maintenance, amplification, or optimal calibration of uncertainty. Berlyne’s (1971) classic inverted-U relationship between arousal and hedonic value, and its computational reformulation by Van de Cruys et al. (2017) as a predictive coding account of aesthetic appreciation, suggest that optimal aesthetic engagement requires a sweet spot between too much and too little prediction error. The sublime, the uncanny, tragic catharsis, avant-garde defamiliarization all involve the tolerance of unresolved or amplified uncertainty. The Distancing-Embracing model (Menninghaus et al., 2017; see also Menninghaus et al., 2019 for a refined formulation) is relevant here: the aesthetic frame creates a safe context in which negative emotions can be embraced rather than avoided, potentially training the allostatic system to tolerate wider state fluctuations without triggering chronic anticipatory activation. This capacity to dwell productively in uncertainty may constitute a form of allostatic flexibility training that purely goal-directed activities cannot provide.

These three features are not absolute but relative: arts engagement provides more of each than most other everyday activities, while other activities may provide more of specific features. The prediction is not that arts engagement produces larger effects on any single biomarker than a domain-specific intervention (sports might produce larger cardiovascular improvements, for instance), but that it produces a more broadly distributed effect across the allostatic load composite: broad-spectrum, in effect, rather than narrow-spectrum, because it engages multiple levels of the predictive hierarchy simultaneously. On this view, the framework predicts that any structured agentic activity possessing the three features identified above (multi-hierarchical engagement, epistemic dominance, uncertainty tolerance) should produce qualitatively similar allostatic effects, regardless of whether it is conventionally classified as “art.” The empirical question is whether activities conventionally grouped as “arts” tend to combine these features more reliably than other activities, a hypothesis that the framework renders testable.

*Boundary conditions and individual differences*. The framework predicts that arts engagement is beneficial only when it provides successful active inference experiences. An arts activity that produces uncontrollable uncertainty, humiliation, or persistent failure would increase, not decrease, allostatic load. Individuals with severe allostatic dysregulation may lack the capacity to engage productively, creating a potential floor effect. A related question is how individuals whose generative models have learned that action is uninformative, i.e., the computational signature of depression described in Section 3, can be motivated to engage at all. Several features of arts contexts may lower this threshold: the low pragmatic stakes reduce the cost of failed predictions compared to real-world action; social scaffolding in group settings allows others to model successful engagement; and the novelty of the aesthetic context may bypass learned helplessness models that are domain-specific rather than global, activating exploratory behavior in a domain that the system has not yet learned to suppress. The framework predicts that individuals with moderate allostatic dysregulation and intact capacity for voluntary engagement should show the largest effects.

An important boundary condition concerns the distinction between active and receptive engagement. The framework emphasizes agentic, active engagement as the primary therapeutic mechanism, and the implications section notes that passive consumption without interpretive engagement may be insufficient for allostatic recalibration. However, the epidemiological literature documenting arts-health associations (Fancourt and Steptoe, 2019; Fancourt and Finn, 2019) includes substantial passive engagement (attending concerts, visiting museums, watching films). This apparent discrepancy can be resolved within the predictive processing framework: from the perspective of active inference, perception is never truly passive. A listener at a concert is continuously generating predictions about melodic trajectory, harmonic progression, and dynamic contour; a museum visitor is actively constructing meaning from visual stimuli. What distinguishes “active” from “passive” engagement, in computational terms, is not the presence or absence of predictive processing (which is ubiquitous) but the depth of hierarchical engagement, the degree of epistemic motivation, and the precision of the prediction errors generated. Receptive aesthetic engagement is predicted to produce allostatic effects through the same mechanism as active participation, but at a lower intensity and across fewer hierarchical levels, consistent with the epidemiological finding that both receptive and participatory engagement are associated with health benefits, though participatory engagement tends to show stronger associations (Fancourt and Steptoe, 2019).

*Conscious access and voluntary control*. A question raised by the foregoing is the extent to which the processes invoked here are conscious, and the extent to which they can be voluntarily influenced. The framework’s answer is that the core computational operations, precision weighting, prediction error propagation, and interoceptive inference, are predominantly non-conscious and subpersonal: a listener does not deliberately set the gain on auditory prediction errors, and a participant cannot directly exert a change in diurnal cortisol slope. What is available to voluntary control is not the machinery itself but its inputs and contexts, and this is precisely where arts engagement works its way through. Three points of voluntary entry can be identified. The first is the decision to engage at all, and the selection of one activity over another, which determines which hierarchical levels are exercised (Section 8). The second is the direction of attention, which is partly voluntary and which modulates precision: attending to the breath, to a melodic line, or to a partner’s movement raises the precision of prediction errors in that domain, so that deliberate attentional engagement is a route by which a person can bias otherwise automatic precision allocation. The third is interpretive effort, the voluntary elaboration of meaning that recruits higher levels of the hierarchy (L3) and sustains engagement over time. On this account, the therapeutic agency of the participant operates through these accessible levers: choice, attention, and interpretation, which then propagate downward to recalibrate processes that are not themselves under direct voluntary control. This also clarifies the active-receptive distinction drawn above: what distinguishes more from less therapeutically potent engagement is largely the degree to which these voluntary levers are deployed, rather than a categorical difference in the underlying mechanism.

## 8 Domain-specific active inference training across art forms

Whereas the preceding section identified features that are common to arts engagement generally, different art forms emphasize different components of the active inference hierarchy, providing domain-specific engagement that may have distinct physiological correlates.

*Music and temporal prediction*. Musical experience is fundamentally an exercise in temporal active inference: continuous generation of predictions about melodic contour, harmonic progression, rhythmic pattern, dynamic trajectory, among others (Huron, 2006; Koelsch et al., 2019). Vuust et al.’s (2022) comprehensive review confirms that prediction errors in music activate fronto-temporal predictive processing networks. Cheung et al. (2019) showed that musical pleasure is jointly predicted by uncertainty and surprise, consistent with the optimal prediction error account rather than simple error minimization. Brattico et al. (2013) showed that musical aesthetic judgments engage both cognitive and affective evaluation networks. Salimpoor et al. (2011) provided direct neurochemical evidence that the reward of musical experience is linked to predictive processing rather than to the sensory stimulus per se. Subsequent work (Salimpoor et al., 2013) showed that the monetary value that individuals assign to novel music tracks predicts the degree of functional connectivity between the auditory cortex and the mesolimbic reward system, reinforcing the link between aesthetic prediction and dopaminergic valuation. The multi-timescale structure of music (beat-level entrainment through phrase-level expectation to form-level narrative arc) exercises hierarchical depth in a way that is computationally demanding and affectively rewarding. The specific aesthetic emotions evoked by music such as chills, being moved, or awe (Sachs et al., 2016; Wassiliwizky et al., 2017) may constitute distinctive signatures of successful hierarchical prediction error processing; Schoeller and Perlovsky (2016) have argued that aesthetic chills specifically reflect the resolution of conflicts between competing knowledge structures, providing a direct bridge between the active inference account of curiosity and the phenomenology of aesthetic experience. It should be noted that the chills response shows substantial individual variation and is modulated by personality traits, particularly openness to experience and absorption (Nusbaum and Silvia, 2011), which has implications for the framework’s prediction about individual differences in therapeutic response: individuals with higher trait openness may show stronger precision recalibration effects from musical engagement. This may explain the particularly robust associations between musical engagement and stress-related biomarkers documented in the literature (Fancourt et al., 2016; Koelsch, 2014).

*Visual arts and perceptual inference*. Visual art engagement exercises the predictive system’s capacity to resolve perceptual ambiguity. Art-making involves the active construction of visual representations: predicting how a mark, color, or composition will appear before executing it, then comparing the result against the prediction. Art-viewing, particularly of works that exploit perceptual ambiguity or defamiliarization, requires the viewer to actively construct meaning from underdetermined inputs. Van de Cruys et al.’s (2017) predictive coding account proposes that aesthetic appreciation arises from the fluent processing of stimuli that initially generate prediction errors but then yield to successful model updating, a moment of comprehension that is inherently rewarding. Pelowski et al.’s (2017) Vienna Integrated Model (VIMAP) provides a multi-stage account from automatic perceptual processing through deliberate cognitive evaluation to potential transformational experience, mapping well onto the hierarchical account proposed here. Gallese and Freedberg (2007) have argued that the experience of visual art engages embodied simulation mechanisms: mirror neuron systems and other sensorimotor circuits are activated during the perception of depicted actions, gestures, and even textures, producing a bodily resonance that grounds aesthetic experience in the observer’s own motor and interoceptive repertoire. This embodied simulation account indirectly supports the active inference framework developed here, as it implies that visual art viewing is not a purely cognitive operation but an embodied predictive process that engages L1 (sensorimotor) and L2 (affective) levels of the hierarchy alongside L3 (semantic-interpretive) processing. Vessel et al. (2012, 2013) showed that intense aesthetic experience activates the default mode network associated with self-referential processing and interoception, suggesting that powerful visual art engagement integrates with the organism’s own interoceptive and affective models. Evidence from clinical settings supports the health relevance of these mechanisms: visual arts interventions have been associated with reduced cortisol and improved mood in diverse patient populations (Stuckey and Nobel, 2010), though the specific contribution of perceptual prediction engagement versus non-specific factors remains to be isolated.

*Dance and embodied active inference*. Dance engages the active inference system at its most fundamental level: integrating interoceptive, proprioceptive, and exteroceptive predictions in real-time sensorimotor control (Blaesing et al., 2012). The dancer continuously generates motor predictions, compares them against proprioceptive and visual feedback, and adjusts in real time, directly training the precision of bodily state models. Koch et al.’s (2014) meta-analysis documented significant effects of dance on depression, quality of life, and body image. Christensen et al. (2018) showed that dance expertise modulates interoceptive accuracy, providing direct evidence that embodied arts practice trains the visceral prediction capacities underlying efficient allostatic regulation. When dance involves social synchronization as in partner dance, group choreography, and contact improvisation, it additionally engages social active inference, requiring coordination of one’s own predictions with co-participants’ (Keller et al., 2014). The interoceptive dimension is particularly significant from an allostatic perspective: by training the accuracy of visceral predictions, dance may directly enhance the interoceptive inference on which allostatic regulation depends (Barrett, 2017).

*Fiction and social inference*. Narrative fiction engagement exercises the predictive system’s capacity for social and affective inference (Oatley, 1999; Mar and Oatley, 2008). Readers continuously generate predictions about characters’ mental states and behaviors, testing these against the unfolding narrative. The evidence that reading fiction improves theory of mind (Kidd and Castano, 2013) has faced significant replication challenges (Panero et al., 2016; Samur et al., 2018), and the effect appears smaller and more contingent on text type than initially claimed. However, the neuroscientific evidence for narrative engagement as predictive processing is more robust: Hasson et al.’s (2004) demonstration of inter-subject synchronization during narrative viewing, Nguyen et al.’s (2019) findings on shared neural responses, and Willems et al.’s (2020) account of literary reading as predictive processing all support the view that fiction provides rich contexts for exercising social generative models. The concept of narrative transportation (Green and Brock, 2000), that is, deep immersion that modifies beliefs and potentially physiological states, may reflect the temporary adoption of an alternative generative model that exercises the system’s flexibility. It is worth noting that deep narrative transportation characteristically involves a reduced awareness of one’s own bodily state, which might appear to conflict with the interoceptive mechanism described in Section 9. The resolution is that fiction primarily exercises top-down model flexibility and social prediction rather than interoceptive precision per se; the two mechanisms are complementary, addressing different levels of the same hierarchical system. However, emerging evidence suggests that this dissociation may be less sharp than it initially appears. Lehne and Koelsch (2015) have proposed a general psychological model of tension and suspense showing that narrative engagement produces measurable autonomic modulation, including changes in skin conductance and heart rate, even when subjective bodily awareness decreases. Narrative fiction may therefore modulate interoceptive states even when interoceptive awareness is reduced, which itself constitutes an interesting prediction: the allostatic benefit of fiction may operate partly through autonomic modulation below the threshold of conscious bodily awareness. From an allostatic perspective, fiction provides low-cost exploration of social contingencies that constitute a major source of allostatic burden in human life (Beckes and Coan, 2011). The pathway from fictional social prediction (L3) to inflammatory markers is hypothesized to operate through two routes: first, through the social buffering mechanism, where vicarious social engagement during fiction reading partially activates the neural substrates of social proximity (Beckes and Coan, 2011), reducing the metabolic cost of social uncertainty, though the magnitude of this effect is expected to be smaller than that produced by actual social co-regulation; and second, through the autonomic modulation documented by Lehne and Koelsch (2015), which provides a direct visceral pathway from narrative engagement to allostatic regulation, bypassing the need for conscious interoceptive awareness.

The critical implication of this domain-specific analysis is that different art forms engage different hierarchical levels with different degrees of specificity: intervention design should match the art form to the specific level of predictive processing that is most compromised in a given individual or population, and the diversity of available art forms is itself a therapeutic resource. This claim is currently supported by the computational logic of the framework and by the neuroimaging evidence reviewed above, but direct comparative evidence for differential effectiveness of different art forms on specific biomarkers does not yet exist. Generating such evidence is a priority for the empirical research program proposed in Section 10.

## 9 Allostatic recalibration through the arts: four interconnected mechanisms

If arts engagement exercises active inference capacities, and if allostasis operates through the same predictive architecture, then the pathway from arts participation to physiological health effects can be specified with considerably more precision than existing accounts allow. Four interconnected mechanisms are proposed.

*Prediction-action loop restoration*. The most direct mechanism is the re-establishment of functional anticipation-action-outcome contingencies. In chronic stress, learned helplessness, or trauma, the active inference system has learned that action does not reliably produce predicted outcomes as the precision on action-dependent prediction errors has collapsed. Arts engagement, by providing settings in which creative action consistently produces observable, meaningful consequences, directly counteracts this learned contingency failure. Each completed creative act constitutes a successful predict-act-confirm cycle of the kind described in Section 5. Over repeated engagements, this accumulation may gradually rebuild the precision assigned to action-dependent prediction errors, restoring the organism’s expectation that its actions can change its world. The dopaminergic system, which, on the active inference account, encodes the precision of reward prediction errors and is blunted in depression (Treadway and Zald, 2011), may be a particular target of this recalibration.

*Flexible allostatic set-points*. Different arts activities require different physiological configurations: the concentrated stillness of close reading, the heightened arousal of performance, the relaxed alertness of improvisation, the rhythmic entrainment of musical ensemble. Regular participation trains state-switching, i.e., efficient movement between different autonomic, neuroendocrine, and metabolic configurations. Chronic stress produces rigid, high-activation set-points that resist adjustment (McEwen, 2007). By exercising the system across different configurations, arts engagement may restore the dynamic range characterizing healthy allostatic function. The capacity to tolerate amplified uncertainty in aesthetic experience may be particularly important here, enabling the system to undergo large-amplitude state transitions and return to baseline. Heart rate variability and baroreceptor reflex sensitivity, as indices of autonomic flexibility, provide readily measurable proxies for this mechanism (Thayer et al., 2012).

*Enhanced interoceptive inference*. Embodied arts engagement as manifested in dance, theater, vocal performance, and to varying degrees all arts practices involving bodily sensation and expression, trains the accuracy of interoceptive prediction. Interoceptive accuracy is reduced in conditions associated with allostatic dysregulation, including depression, anxiety, and chronic stress (Paulus and Stein, 2010; Khalsa et al., 2018). Christensen et al.’s (2018) finding that dance expertise enhances interoceptive accuracy provides direct empirical support. As they draw sustained attention to bodily states in contexts of safe exploration, embodied arts practices may enhance the precision of interoceptive predictions, leading to more accurate visceral state estimation and more efficient autonomic regulation. Improved interoceptive inference would also produce more accurate emotional experience (i.e., a more faithful running commentary on allostatic state), which in turn supports more adaptive behavioral regulation.

*Social allostatic regulation*. Many arts activities are inherently social, involving joint attention, collaborative creation, and synchronized action. Social interaction is itself a form of active inference: each participant must model the other’s predictions, intentions, and likely responses (Friston and Frith, 2015). Feldman’s (2017) biobehavioral synchrony model describes how social coordination produces physiological co-regulation through synchronized autonomic, hormonal, and neural responses, a mechanism mapping naturally onto the active inference framework as the alignment of generative models between agents. The social baseline theory (Beckes and Coan, 2011; Coan et al., 2006) proposes that attuned co-regulators reduce the metabolic cost of uncertainty management. Collective arts engagement in group singing, ensemble music, or collaborative theater may be particularly effective because it simultaneously exercises individual active inference capacities and provides social co-regulation that directly buffers allostatic load. Synchronized physiological responses during choir singing (Müller and Lindenberger, 2011) and the social bonding effects of group singing (Kreutz, 2014) support this mechanism. Social prescribing programs, which connect individuals with community arts activities, may derive a substantial part of their effectiveness from this social co-regulation pathway in addition to the arts content per se, a hypothesis that is testable by comparing individual versus group delivery of otherwise identical arts interventions.

The four mechanisms are presented separately for analytical clarity, but the framework hypothesizes deep relationships among them. Prediction-action loop restoration (mechanism 1) is hypothesized to be foundational for active, participatory arts engagement: without at least a minimal recovery of the expectation that action can produce predicted outcomes, the other mechanisms cannot be fully engaged in agentic contexts, since active participation itself depends on a threshold level of agentic engagement. This sequential dependency applies most clearly to participatory arts engagement; receptive engagement (concert attendance, museum visits, fiction reading) may activate mechanisms 2 and 3 through perceptual predictive processing without requiring prior restoration of action precision, consistent with the argument in Section 7 that perception is itself a form of active inference. The foundational role of mechanism 1 is therefore hypothesized to be graded rather than absolute: it is a prerequisite for the full engagement of the other mechanisms at the intensities that are characteristic of active participation, but receptive engagement may produce attenuated effects on mechanisms 2 and 3 independently.

This hypothesis is derived from the computational logic of the framework (the other mechanisms require active engagement, which presupposes a minimal level of action precision) rather than from existing empirical evidence, and should be treated as a structural prediction to be tested. Flexible set-points (mechanism 2) and enhanced interoceptive inference (mechanism 3) are hypothesized to operate in parallel, as partially independent consequences of sustained arts participation, each contributing to allostatic recalibration through a distinct route (autonomic flexibility and visceral prediction accuracy, respectively). Social co-regulation (mechanism 4) is hypothesized to moderate the effectiveness of mechanisms 1 through 3: group-based arts engagement should amplify the effects of each individual mechanism by providing the co-regulatory buffering that reduces the metabolic cost of the model updating process itself. This causal architecture generates a specific prediction: individual arts engagement should produce measurable effects through mechanisms 1 through 3 alone, but the addition of social components should produce effects that exceed the sum of social contact and individual arts engagement separately, because social co-regulation reduces the allostatic cost of the model updating that mechanisms 1 through 3 require.

**Figure 2:**
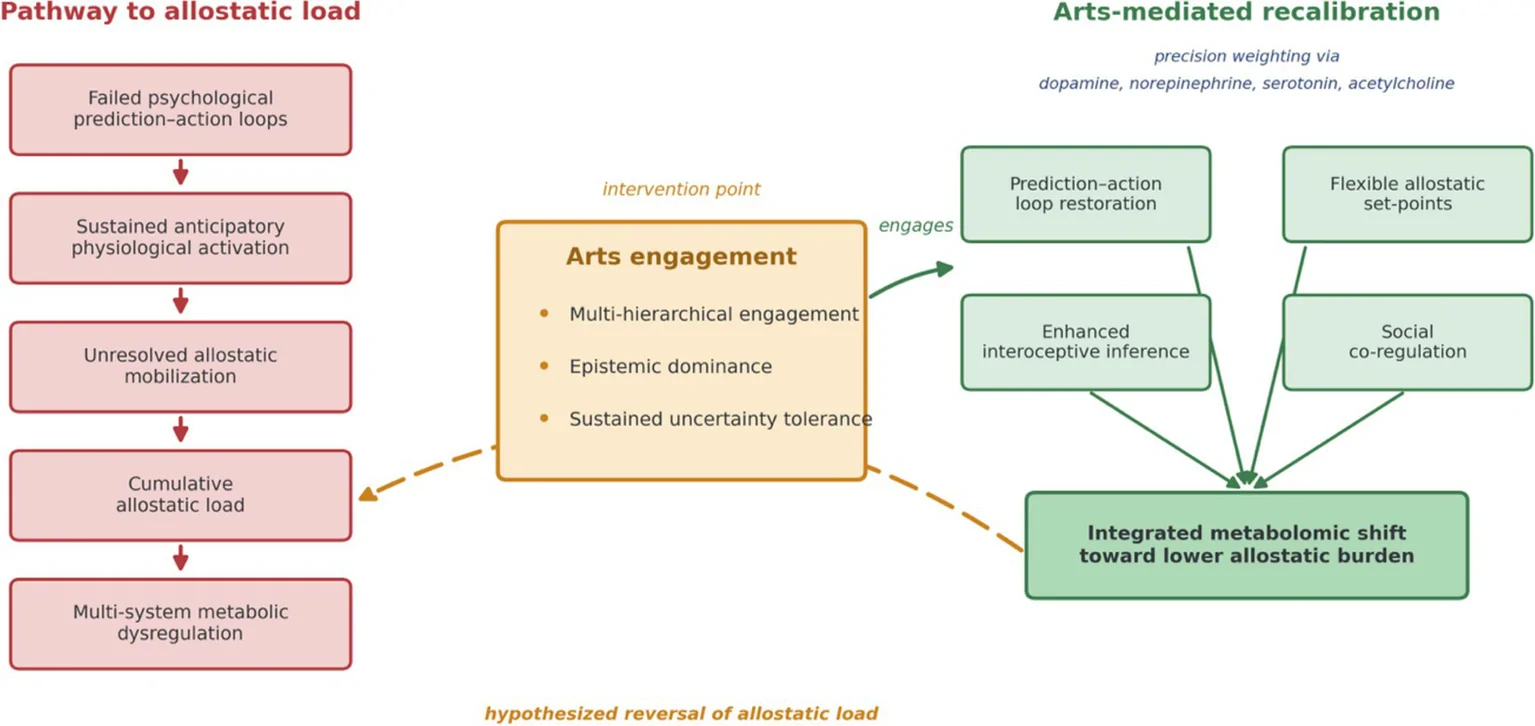
Schematic representation of the proposed framework. Left panel: The cascade from failed active inference to allostatic load (failed psychological prediction-action loops → sustained anticipatory physiological activation → unresolved allostatic mobilization → cumulative allostatic load → multi-system metabolic dysregulation). Center inset: The three distinguishing features of arts engagement as an intervention point (multi-hierarchical engagement, epistemic dominance, sustained uncertainty tolerance). Right panel: The four mechanisms through which arts-mediated active inference restoration produces allostatic recalibration (prediction-action loop restoration, flexible set-points, enhanced interoceptive inference, social co-regulation), converging on an integrated metabolomic shift toward lower allostatic burden. Neuromodulatory systems (dopamine, norepinephrine, serotonin, acetylcholine) that implement precision weighting and are targets of both allostatic dysregulation and arts-mediated recalibration are indicated. Dashed arrows indicate the hypothesized reversal pathway.

Figure 2 offers a schematic representation of the proposed framework.

## 10 Empirical predictions and falsifiability conditions

The framework generates predictions at multiple levels. Critically, these predictions must be distinguishable from what a generic “stress reduction” account would predict, as otherwise the active inference formulation adds no explanatory value. It should be noted at the outset that the predictions below are directional hypotheses derived from the schematic of Section 6; they specify the direction and ordering of expected effects rather than point estimates or precise effect sizes. Translation into quantitative predictions awaits the formal parameterization discussed in Section 6 and acknowledged as a limitation in Section 13. The following predictions are organized around what the framework predicts that simpler accounts do not.

To make these empirical commitments concrete, and to indicate which of them are tractable outside the laboratory, Table 1 brings together the operational measures, study designs, and analyses associated with each construct and mechanism of the framework.

**Table 1:** Operationalization of the framework’s key constructs.

**Target**	**Laboratory or computational measure**	**Lower-burden field measure**	**Suggested design and analysis**
Action precision (π_action_)	Model-based vs. model-free weighting on the two-step task (Daw et al., 2011)	Agency and locus-of-control self-report; task-completion rates	Pre-post with active control; mixed-effects models of within-subject change
Interoceptive precision (π_intero_)	Heartbeat discrimination with confidence ratings (Garfinkel et al., 2015)	Phone-timed heartbeat-counting task (Schandry, 1981)	Pre-post stratified by baseline accuracy; baseline-by-intervention interaction
Social precision (π_social_)	Computational models of joint action and belief updating	Network-size and perceived-support inventories	Individual vs. group delivery contrast; mediation by social measures
Sensory precision (π_sensory_)	MMN and P300 amplitude to deviants (Garrido et al., 2009)	Brief portable-EEG MMN; sensory-sensitivity questionnaires	Trained vs. untrained deviant domains; domain-by-time ANOVA
Allostatic load (composite)	Full multi-system biomarker panel (Seeman et al., 1997; Juster et al., 2010)	Multi-point home salivary cortisol; resting heart rate and blood pressure; waist-hip ratio	8–12 week intervention with baseline-load strata; composite-index change and stratum interaction
Allostatic prediction error and HPA dynamics	Continuous interoceptive-autonomic mismatch indices	Intra-day salivary cortisol coefficient of variation; ambulatory HRV (wearable or chest strap)	Repeated within-day sampling across weeks; trajectory modeling with non-monotonic (quadratic) tests
Neural prediction-error processing	Task-fMRI three-network convergence; resting-state connectivity; voxel-based morphometry	Research settings only (not low-burden)	Comparison conditions isolating triad components; conjunction and ROI connectivity analyses

The columns give, respectively, a laboratory or computational measure, a lower-burden measure suitable for applied and community settings, and a suggested study design with its primary analytic approach.

*Metabolomic predictions specific to the active inference mechanism*. *Temporal dynamics.* A generic stress reduction account predicts simple dose–response: more sessions, more benefit, monotonically. The active inference account predicts a qualitatively different trajectory. Because the mechanism involves model updating (the gradual rebuilding of generative model parameters), the framework predicts an initial period of increased metabolic variability (reflecting the metabolic cost of model updating as the system explores new parameter configurations) followed by progressive stabilization at lower allostatic load levels. Specifically, the predicted variability increase concerns the diurnal cortisol profile: the intra-individual coefficient of variation (CV) of salivary cortisol sampled at multiple time points across each assessment day is predicted to increase during the early weeks of a regular arts engagement program (reflecting HPA axis reorganization during active model updating), then progressively decrease toward a CV lower than baseline as the intervention continues (reflecting stabilization at a new, more efficient allostatic configuration). This prediction is specific to cortisol variability; HRV is predicted to show a more monotonic improvement, since HRV reflects autonomic flexibility rather than the model updating process per se. The magnitude and precise timing of the cortisol CV increase cannot be derived from the current specification and must be determined empirically. For purposes of statistical power analysis and study design, analogous intervention timescales from mindfulness-based stress reduction (Krygier et al., 2013) and psychotherapy (Steudte-Schmiedgen et al., 2016) suggest that effects may become detectable at approximately 4 weeks and stabilize by 8 to 12 weeks, but these estimates should be understood as design heuristics drawn from related intervention literatures rather than as predictions of the framework itself. The initial variability increase should be distinguishable from the acute stress of novelty (performance anxiety in beginners) by its temporal profile: novelty-related cortisol elevation would be expected to peak early and decline as familiarization occurs, whereas the model-updating signature is an increase in variability (not necessarily in mean level) that emerges after initial familiarization, reflecting the metabolic cost of active generative model reorganization.

A bridging assumption is required here: the claim that computational model updating in cortical circuits produces a measurable signature in salivary cortisol variability depends on the well-established coupling between sustained cognitive-affective processing and HPA axis activation (Dickerson and Kemeny, 2004). Any novel, socially evaluative, or effortful task can acutely elevate cortisol. The framework’s prediction is not that cortisol variability increases per se (which would be a generic cognitive load effect), but that the temporal profile of the variability increase is distinct: a generic cognitive load effect should peak when task demands are highest (typically early in the intervention, during the unfamiliar initial sessions) and decline as habituation occurs, whereas the model-updating prediction is that variability should remain elevated or increase further after initial habituation, reflecting the ongoing metabolic cost of generative model reorganization that begins only once the surface-level novelty stress has subsided. This temporal dissociation, if observed, would constitute evidence for model updating beyond generic task demand, though it must be acknowledged that the two processes may overlap substantially in practice and that distinguishing them empirically will require carefully timed repeated assessments.

*Differential sensitivity of allostatic load components.* Using the operationally defined allostatic load composite (Juster et al., 2010; Seeman et al., 1997), the framework predicts that neuroendocrine components (cortisol pattern, DHEA-S ratio) should respond earliest, reflecting direct HPA axis recalibration through restored predict-act-confirm contingencies. Immune-inflammatory markers (CRP, IL-6) should respond on a slower timescale, reflecting downstream metabolic consequences of sustained neuroendocrine recalibration. The predicted ordering reflects a causal sequence rather than merely a difference in measurement resolution: cortisol normalization is hypothesized to produce downstream immune-inflammatory changes through the well-established glucocorticoid regulation of pro-inflammatory cytokine expression (Silverman and Sternberg, 2012). This causal claim is testable through mediation analysis: change in diurnal cortisol slope during the early intervention period should statistically mediate subsequent change in CRP and IL-6 during the later intervention period, though the specific timescales at which these effects become detectable must be determined empirically rather than specified *a priori* by the framework.

The kynurenine pathway, which diverts tryptophan metabolism from serotonin synthesis toward neurotoxic metabolites under chronic inflammation (Dantzer et al., 2008), provides a specific molecular link in this cascade: as a downstream consequence of the sustained immune-inflammatory activation characteristic of allostatic load, serotonergic function is degraded, potentially disrupting the precision of prior beliefs and temporal prediction that serotonin is hypothesized to regulate (Carhart-Harris and Friston, 2019). On this account, the kynurenine-mediated depletion of serotonin under chronic stress does not merely produce “low mood” but specifically impairs the system’s capacity to maintain appropriately weighted priors across time, contributing to the hopelessness and foreshortened future characteristic of depression. Normalization of immune-inflammatory markers should precede and predict subsequent improvements in serotonergic function. Cardiovascular and metabolic components (blood pressure, lipid profiles, glycosylated hemoglobin) should respond latest.

It should be acknowledged that this kynurenine prediction is not specific to arts engagement or to the active inference mechanism in isolation: any intervention that reduces chronic inflammation (exercise, anti-inflammatory medication, psychotherapy) would produce the same downstream effect. What is framework-specific is the predicted temporal ordering of the entire cascade (neuroendocrine first, immune-inflammatory second, kynurenine-serotonergic third, cardiovascular-metabolic last) and the predicted non-monotonic dynamics within the earliest component. This ordered cascade distinguishes the framework from a generic wellbeing account, which would predict simultaneous improvement. The non-linear character of HPA axis dysregulation (Miller et al., 2007; Heim et al., 2000) means that the cortisol prediction is specifically that diurnal cortisol slope should steepen toward normative values, and whether this involves reducing or increasing absolute cortisol depends on the individual’s baseline pattern.

*Art-form-specific metabolomic signatures.* If different art forms engage different primary levels of the predictive hierarchy, they should produce partially distinguishable biomarker profiles, with relative rather than absolute specificity since all art forms engage multiple hierarchical levels, and the neurochemical systems involved in precision weighting are not neatly segregated by level. The prediction is directional: music engagement, emphasizing temporal prediction and affective processing, should produce relatively stronger effects on dopaminergic metabolites and endocannabinoids. Dance, emphasizing interoceptive-proprioceptive integration, should produce relatively stronger effects on autonomic markers (heart rate variability, catecholamine metabolites) and interoceptive accuracy. Narrative fiction, emphasizing social prediction, should show its primary metabolomic impact on oxytocin-related pathways and inflammatory markers (through social buffering mechanisms). Conversely, fiction should produce relatively weaker effects on interoceptive accuracy and autonomic markers compared to dance, reflecting its primary engagement with top-down social prediction rather than visceral-proprioceptive inference (see Section 8). These partially distinguishable signatures constitute a strong test of the multi-hierarchical account.

*Neuroimaging predictions*. The existing neuroaesthetics literature provides a substantial foundation. Vessel et al. (2012, 2013) showed default mode network activation during intense aesthetic experience. Vuust et al. (2022) documented predictive processing network engagement during music. Chatterjee and Vartanian (2014) identified an aesthetic triad of sensory-motor, emotion-valuation, and meaning-knowledge systems. Kleckner et al. (2017) mapped a large-scale allostatic-interoceptive network encompassing anterior insula, anterior cingulate, amygdala, hypothalamus, and ventromedial prefrontal cortex.

The present framework predicts that effective arts engagement is characterized by the simultaneous activation of (a) exteroceptive predictive processing networks in sensory cortices, (b) interoceptive prediction error circuitry centered on the anterior insula, and (c) reward prediction error signaling in the ventral striatum. This three-network convergence hypothesis is testable using task-based fMRI with comparison conditions that selectively engage subsets of the triad. For example, an adaptive cognitive training task with performance feedback, such as an n-back working memory paradigm with points and graded difficulty (engaging exteroceptive prediction and reward, but with minimal bodily-affective engagement) would test whether component (b) distinguishes arts from non-arts structured activities. A physically demanding but unrewarding task, such as repetitive movement to a metronome, would engage (a) and (b) without (c), testing the contribution of aesthetic reward. Passive viewing of well-structured but affectively neutral visual stimuli, such as simple geometric patterns, would engage (a) without substantial (b) or (c), distinguishing low-level perceptual processing from the active, uncertainty-laden engagement that the framework predicts is necessary for allostatic recalibration.

Beyond task-based activation, the framework generates structural predictions. Longitudinal arts participation should enhance resting-state functional connectivity within the allostatic-interoceptive network identified by Kleckner et al. (2017), distinguishable from connectivity changes produced by exercise or meditation. If the mechanism operates through precision recalibration at the insular cortex, sustained arts engagement may also produce detectable changes in gray matter volume or cortical thickness in the anterior insula and ventromedial prefrontal cortex, that is, regions where structural plasticity has been documented following other forms of sustained training (e.g., mindfulness meditation, musical expertise). Voxel-based morphometry comparing long-term arts practitioners with matched controls, complemented by longitudinal scanning of intervention participants, would test this prediction.

EEG and MEG approaches offer complementary temporal resolution. The mismatch negativity (MMN) component, which indexes automatic prediction error detection, and the P300, which indexes attentive evaluation of unexpected events (Garrido et al., 2009), should both show modulation during and after sustained arts engagement programs. A particularly informative design would compare MMN responses to prediction violations in arts-relevant domains (e.g., melodic deviants for musicians, visual pattern deviants for visual artists) versus non-trained domains, testing whether arts engagement produces domain-specific or domain-general changes in prediction error processing. Domain-specific enhancement would support the hierarchical specificity argument; domain-general enhancement would suggest a broader recalibration of precision weighting.

*Falsifiability conditions*. The framework would be disconfirmed by the following findings. (a) If controlled arts interventions with confirmed participant engagement produce no detectable change in allostatic load biomarkers over a 12-week period, despite producing subjective wellbeing improvements, this would suggest that health effects bypass the allostatic pathway. (b) If the temporal dynamics of metabolomic change are indistinguishable from those following a matched non-arts active control (e.g., group sports with equivalent social contact and physical activity), the active inference account adds no explanatory value beyond generic stress reduction. (c) If different art forms produce identical metabolomic signatures despite engaging different hierarchical levels, the multi-hierarchical specificity claim is weakened. (d) If individuals with intact active inference capacities (low allostatic load, high interoceptive accuracy) show equivalent metabolomic responses to individuals with compromised capacities, the mechanism does not operate through active inference restoration specifically. In operational terms, the prediction of largest effects in individuals with moderate dysregulation could be tested by stratifying participants by baseline allostatic load index (Juster et al., 2010), with the prediction that the intervention-by-stratum interaction should be largest in intermediate strata. The specific cut points for stratification should be determined by the sample distribution and the clinical characteristics of the population rather than specified *a priori* by the framework. The qualitative prediction is that individuals with very low allostatic load should show ceiling effects, and individuals with severe dysregulation may require adjunctive clinical support before arts engagement becomes effective, but the boundary between “moderate” and “severe” dysregulation is an empirical question that the framework identifies but cannot resolve in advance of parameterization. (e) If neuroimaging fails to reveal three-network convergence during aesthetically engaging arts experiences, the proposed convergence mechanism requires revision. It is worth emphasizing that the framework is not disconfirmed by the finding that non-arts activities also produce allostatic recalibration, as the claim is that the arts constitute a distinctive instance, not the exclusive route. The framework is disconfirmed if arts engagement produces no effects that are quantitatively or qualitatively distinguishable from matched non-arts activities on the specific dimensions predicted above.

## 11 Comparison with alternative frameworks

Several existing frameworks propose mechanisms through which arts engagement may influence health. A systematic comparison clarifies what the active inference account adds and where predictions diverge.

*Relaxation response* (Benson et al., 1974). The relaxation response model proposes that arts engagement, like meditation and yoga, produces health benefits by eliciting a generalized state of reduced sympathetic arousal (decreased heart rate, blood pressure, and cortisol) that counteracts the chronic activation of the fight-or-flight response. The active inference framework diverges in two critical ways. First, it predicts that the temporal dynamics of metabolomic change should be non-monotonic (initial increased variability before stabilization) rather than the simple dose–response that a relaxation account predicts. Second, it predicts that some of the most therapeutically effective aesthetic experiences involve increased, not decreased, arousal (the sublime, catharsis, chills, performance excitement) because the therapeutic mechanism is not arousal reduction but the completion of prediction-action-outcome cycles. The relaxation response cannot explain why high-arousal arts experiences (rock concerts, theater performance, competitive dance) produce health benefits comparable to low-arousal ones (contemplative art viewing, meditative musical listening). The active inference framework can: what matters is not the level of arousal but whether allostatic mobilization is followed by resolution.

*Social bonding and endorphin theory* (Dunbar, 2012; Tarr et al., 2014). This framework proposes that synchronized movement and singing trigger endorphin release through coordinated muscular exertion, producing social bonding that buffers stress through the same mechanisms as primate grooming. Tarr et al. (2014) sreviewed evidence that synchronized movement produces elevated pain thresholds (a proxy for endorphin release) and increased feelings of social closeness, and proposed that this mechanism may have co-evolved with music and dance as a means of maintaining social cohesion in groups larger than those manageable through dyadic grooming. The active inference framework is compatible with this account at the social mechanism level (Section 9): social synchrony and co-regulation are part of the proposed pathway. However, it diverges in scope. The social bonding account cannot explain why solitary arts engagement (reading fiction, painting alone, listening to music in isolation) also produces health benefits, since there is no synchronized movement and no social partner. The active inference account can: solitary arts engagement trains individual active inference capacities (π_action_, π_intero_, π_sensory_) without requiring the social component. The differentiating prediction is that solitary arts engagement should produce metabolomic changes in neuroendocrine and interoceptive markers (cortisol, HRV) even without changes in endorphin-related or oxytocin-related pathways.

*Emotional regulation framework* (Gross, 2015; Koelsch, 2014). This framework proposes that arts engagement improves health by providing opportunities for emotional regulation: reappraisal, distraction, catharsis, emotional expression. The active inference account encompasses emotional regulation as one consequence of improved interoceptive inference (π_intero_) and more accurate allostatic prediction, but it offers a more fundamental mechanism: emotional regulation is effective *because* the underlying generative model is better calibrated, not the other way around. The differentiating prediction concerns the direction of causality: the emotional regulation account predicts that interventions explicitly teaching regulation strategies should be more effective than equivalent arts engagement without regulation instruction, whereas the active inference account predicts that the act of engaging in the arts (generating predictions, testing them, and experiencing resolved or optimally calibrated prediction errors) is itself the mechanism, regardless of whether explicit regulation strategies are taught.

*Broaden-and-build theory* (Fredrickson, 2001). Broaden-and-build proposes that positive emotions produced by arts engagement broaden momentary thought-action repertoires and build durable personal resources. The active inference framework converges in predicting that positive affect during arts engagement is functional rather than epiphenomenal, but it provides a more specific mechanism: positive affect in aesthetic experience is the felt correlate of successful prediction error resolution (Van de Cruys and Wagemans, 2011), not a general mood state that indiscriminately broadens cognition. The differentiating prediction concerns negative aesthetic experiences: broaden-and-build predicts that arts experiences generating negative emotions (tragic drama, disturbing visual art, dissonant music) should be less health-promoting than positive ones, because negative emotions narrow rather than broaden thought-action repertoires. The active inference account predicts the opposite: *provided that the aesthetic frame is maintained* (Menninghaus et al., 2017), arts experiences involving negative emotions may be *more* effective at training allostatic flexibility, because they exercise the system’s capacity to undergo large-amplitude state transitions and return to baseline. Comparing the health effects of positive versus negative arts experiences with equivalent engagement levels would directly test this divergence.

*Polyvagal theory* (Porges, 2007). Porges’ polyvagal theory, which is currently under increasingly critical scrutiny (Grossman, 2023), proposes that the myelinated vagus (“smart vagus”) supports the social engagement system, i.e., the neural platform for calm social interaction, vocalization, and facial expression, and that arts activities, particularly singing and group music, promote health by activating this system. Despite the current controversy on its validity, it may nonetheless be useful to consider polyvagal theory here as a conceptual alternative to assess the impacts of the arts through vagus-related pathways. The active inference framework is compatible at the neural implementation level: the vagal pathways Porges describes may be part of the substrate through which social active inference produces allostatic co-regulation. Vickhoff et al.’s (2013) demonstration that singing enhances vagal tone through respiratory sinus arrhythmia provides empirical support for both frameworks. However, the active inference account is broader: it explains why non-social, non-vocal arts (visual arts, solitary reading, instrumental practice) also produce health effects, which polyvagal theory in its strict formulation cannot. The differentiating prediction is that arts engagement should modulate not only vagal tone but also the precision parameters governing non-social prediction and the broader allostatic-interoceptive network (Kleckner et al., 2017) rather than only the vagal-social engagement circuitry.

*The 50-mechanism taxonomy and the “arts exposome”* (Fancourt et al., 2026). The most comprehensive recent mapping of arts-health mechanisms is Fancourt et al.’s (2026) identification of 50 distinct causal processes spanning emotion, cognition, sense of self, arousal and stress, inflammatory and epigenetic markers, social connections, and behavior. That taxonomy explicitly adopts a complex adaptive systems perspective, concluding that no single mechanism predominates and that the interconnectivity of mechanisms must be considered to understand how they act in synergy. The active inference framework proposed here offers a candidate computational architecture for this integration. Of the 50 mechanisms identified by Fancourt et al. (2026), the majority can be mapped onto specific levels of the hierarchical generative model and specific precision parameters: dopamine release and reward network activation (action selection, π_action_), enhanced predictive coding and cognitive flexibility (L2-L3), improved emotion regulation and interoceptive processing (L2, π_intero_), attentional redirection, attention restoration, and mindfulness-like states (L1-L2, π_sensory_), behavioral synchrony and social brain connectome engagement (L3, π_social_), and adaptive allostasis and autonomic tone regulation (L4). The active inference framework does not replace the 50-mechanism taxonomy but proposes that these mechanisms are not independent causal processes acting in parallel; rather, they are outputs of a single generative architecture, the hierarchical predictive processing system described in Section 6, observed through different empirical windows. Fancourt et al.’s (2026) “arts exposome” concept, which reconceptualizes arts engagement not as isolated interventions but as a totality of cumulative exposures to active ingredients across daily life, is also compatible with the present framework: cumulative arts exposure, on the active inference account, produces its effects through the ongoing calibration and maintenance of generative model parameters, with different art forms and modes of engagement (active participation versus passive reception, high versus marginal attention) contributing differentially to the precision parameters at different hierarchical levels.

This systematic comparison reveals that the active inference framework does not replace these alternative accounts but offers an integrating framework within which each can be situated. Social bonding, emotional regulation, relaxation, positive emotion, and vagal activation, as well as the complex taxonomy of mechanisms identified by Fancourt et al. (2026) may all contribute to arts-health effects, but the active inference framework provides the computational architecture that explains *why* and *how* each contributes, identifies conditions under which each should dominate, and generates predictions that none of the alternative accounts alone can produce. The critical differentiating prediction is the non-monotonic temporal dynamics of metabolomic change: no alternative account predicts initial increased variability, because none invokes model updating as the mechanism.

## 12 Developmental and lifespan perspectives

The framework generates specific predictions about the interaction between arts engagement and developmental timing that go beyond the observation that “early intervention is important.” These predictions follow from the developmental neuroscience of allostatic programming and from the age-dependent plasticity of the predictive hierarchy.

*Critical periods for allostatic set-point calibration*. The evidence on early-life programming of the HPA axis (Lupien et al., 2009; Heim et al., 2000) and the biological embedding of adversity (Hertzman, 2012; Shonkoff et al., 2012) establishes that allostatic set-points are shaped during sensitive periods of neural development, particularly during the first decade of life and again during adolescence. The adverse childhood experiences (ACE) literature documents dose–response relationships between early adversity and adult allostatic load (Danese and McEwen, 2012). Within the present framework, this developmental sensitivity reflects the fact that the generative model’s prior parameters, including the precision weights governing allostatic regulation, are established during these periods and become progressively more difficult to modify as the model consolidates. The framework predicts that arts engagement during these sensitive periods should produce qualitatively different and potentially larger effects on allostatic calibration than adult participation. The basis for this prediction is not simply greater neural plasticity in general, but the specific maturation sequence of the hierarchical levels described in Section 6. Sensorimotor prediction (L1) consolidates earliest, perceptual-affective processing (L2) next, and narrative-social prediction (L3) matures latest, continuing into adolescence and early adulthood as prefrontal circuits reach functional maturity (Casey et al., 2008). This maturation sequence implies that arts engagement during childhood, when L1 and L2 are still being calibrated, should produce its strongest effects on interoceptive and sensorimotor precision (π_intero_, π_sensory_), while arts engagement during adolescence, when L3 is undergoing its most rapid development, should produce its strongest effects on social precision (π_social_) and top-down model flexibility. The developing predictive hierarchy is not simply more plastic than the adult hierarchy; it is differentially plastic at different levels at different times, and the art forms most effective at each developmental stage should be those that target the levels currently undergoing calibration. Regular arts engagement during childhood and adolescence may contribute to the establishment of more flexible and efficient allostatic set-points (higher baseline π_action_, more accurate π_intero_, and more calibrated π_sensory_) with protective effects extending across the lifespan. This prediction is testable through longitudinal studies tracking allostatic load biomarkers in cohorts with varying levels of childhood arts engagement, controlling for socioeconomic confounds.

*Adolescence as a second sensitive period*. Adolescence involves a major reorganization of the prefrontal-subcortical circuits that implement both active inference and allostatic regulation (Casey et al., 2008). The heightened reward sensitivity and exploratory behavior characteristic of adolescence may represent an evolved mechanism for recalibrating the generative model to the specific social and environmental niche the individual will occupy in adulthood. Arts engagement during this period when the system is both maximally plastic and maximally vulnerable may be particularly effective at training the precision parameters governing allostatic flexibility. The well-documented associations between arts participation in adolescence and improved mental health outcomes in young adulthood (Bone et al., 2023) are consistent with this prediction, though again longitudinal studies with biological measures are needed to test the allostatic mechanism specifically.

*Aging and allostatic maintenance*. At the other end of the lifespan, the framework predicts that arts engagement serves an allostatic maintenance function. Age-related decline in predictive processing capacity (reduced precision, less flexible model updating, diminished reward sensitivity) mirrors the multi-level precision degradation described in Section 3 for chronic stress. The epidemiological evidence linking cultural participation in older adults to reduced dementia risk, lower mortality, and better cognitive function (Fancourt and Steptoe, 2019) may reflect the protective effects of sustained active inference training on allostatic regulation. The framework predicts that the most effective arts engagement for older adults should specifically target the precision parameters most affected by aging, likely π_action_ (motivational engagement) and π_social_ (social connectedness), and that the protective effects should be mediated by allostatic load biomarkers.

These developmental and lifespan predictions carry substantial implications for educational and public health policy. If childhood arts engagement calibrates allostatic set-points during sensitive periods, then arts education is not merely a cultural amenity but a population-level investment in allostatic health, one whose benefits compound across the lifespan.

## 13 Limitations

Several important limitations must be acknowledged. First, the framework’s specific predictions remain largely untested. The metabolomic and neuroimaging agenda outlined above constitutes a research program, not a body of confirmed findings. The existing evidence base for arts and health is substantial but faces significant methodological challenges: small sample sizes, lack of blinding (participants know they are in an arts intervention), heterogeneous interventions making cross-study comparison difficult, publication bias toward positive results, and the difficulty of constructing adequate active control conditions (Sheppard and Broughton, 2020; Bone et al., 2023). These are the same weaknesses that have led some commentators to question whether arts-based interventions have yet demonstrated genuine health effects (Skov and Nadal, 2025; Clift, 2020); the framework advanced here does not resolve them, but it specifies the mechanistic predictions and measurement targets against which better-controlled studies could be designed.

In addition, arts engagement is correlated with socioeconomic status, education, social network size, physical activity, and health behaviors. Even in randomized trials, arts programs may produce effects through social contact, physical activity (especially dance), routine structure, novelty, or expectancy effects rather than the specific computational mechanism proposed. Isolating the active inference pathway would require dismantling designs that systematically vary the proposed active ingredients (multi-hierarchical engagement, epistemic framing, uncertainty tolerance) while controlling non-specific factors, combined with mediation analyses incorporating computational measures such as interoceptive accuracy, prediction error sensitivity (indexed by mismatch negativity amplitude), or model-based decision-making parameters. It should be acknowledged that designing such dismantling studies for arts interventions faces unique practical challenges: the three distinguishing features identified in Section 7 are not easily separable in practice, since an arts activity stripped of epistemic motivation or uncertainty tolerance may no longer function as arts engagement in any recognizable sense. Innovative experimental designs, perhaps comparing structurally similar activities that vary in their degree of aesthetic framing, will be needed.

Moreover, the active inference framework, despite its explanatory power, faces the challenge of operationalization. The insights offered here require translation into formal computational models with specific parameterizations before they can generate predictions precise enough for rigorous quantitative testing. Section 6 has identified the empirical measures that could operationalize the key precision parameters (e.g., model-based decision tasks for π_action_, heartbeat detection for π_intero_) and has provided the expected free energy decomposition. However, a fully parameterized generative model that could be fitted to individual participant data and used to derive quantitative predictions remains an open project. Ongoing work in computational psychiatry (Smith et al., 2021) provides a methodological template, but application to arts engagement contexts must still be developed.

A further limitation concerns cultural variation. Aesthetic responses, the forms of arts engagement available, and the social meanings attached to artistic participation vary substantially across cultures. The framework as presented is formulated at the level of computational architecture, which is assumed to be species-universal, but the specific arts practices that effectively engage the predictive hierarchy, and the social contexts in which they do so, are culturally shaped. Whether the three distinguishing features identified in Section 7 operate identically across cultural contexts, or whether cultural variation in aesthetic norms and practices modulates the allostatic pathway, is an empirical question that the present review cannot answer. Cross-cultural research comparing the allostatic effects of arts engagement in populations with different aesthetic traditions and different baseline cultural participation patterns would be needed to address this gap.

Finally, the distinction between acute and chronic effects requires clarification. A single arts session may produce transient mood improvement and cortisol reduction without affecting allostatic set-points. The framework’s claims about allostatic recalibration imply sustained participation over the timescale of model updating (provisionally estimated at 8–12 weeks). Whether there is a minimum effective dose, whether benefits are cumulative or subject to diminishing returns, and how the time course relates to generative model updating dynamics are empirical questions that the framework raises but cannot yet answer.

## 14 Implications

At the level of basic neuroscience, the framework proposes that aesthetic experience is a functional expression of the brain’s predictive architecture rather than an epiphenomenal ‘cheesecake for the mind’ (Pinker, 1997). If the brain maintains its viability through hierarchical active inference, then the drive to seek settings providing rich, graded, multi-scale opportunities for prediction testing (that is, the drive toward aesthetic engagement) is a deep organismic tendency. The ubiquity of art across human cultures and the substantial metabolic resources devoted to artistic activities become intelligible as expressions of a computational imperative: maintaining the generative models on which physiological regulation depends.

For computational psychiatry, the framework suggests that arts-based interventions, when properly designed, target the computational processes whose failure underlies stress-related pathology. Effectiveness should depend not merely on relaxation-inducing properties but on the provision of settings with clear action-outcome contingencies, controllable uncertainty, multi-modal feedback, epistemic motivation, and multi-timescale engagement. Concretely, this translates into design principles: activities should offer graduated challenge calibrated to the participant’s current capacity (avoiding both boredom and overwhelm); they should support interpretive freedom rather than prescribing correct answers; they should engage at least two sensory modalities; and when possible, they should involve social coordination with other participants. Programs that lack these features such as low-engagement aesthetic exposure without sustained interpretive processing, prescriptive activities that eliminate uncertainty, or competitive formats that reintroduce performance anxiety, may produce transient affective improvement without addressing the underlying computational deficit. It must be emphasized, however, that arts-based interventions are positioned here as a complement to, not a replacement for, established pharmacological and psychotherapeutic treatments; the framework does not imply that aesthetic engagement is sufficient to treat diagnosed disorders, and the magnitude of any clinical effect in patient populations remains to be established. In individuals with severe allostatic dysregulation, arts engagement is likely to be most appropriate as an adjunct measure delivered alongside, and under the coordination of, standard clinical care. For practitioners delivering arts activities in clinical or community settings, the most accessible of the measures summarized in Table 1, namely multi-point home collection of salivary cortisol, ambulatory heart rate variability from a consumer wearable or chest strap, and a brief heartbeat-counting task, together provide a feasible, low-burden battery for tracking change in allostatic and interoceptive markers without specialized laboratory infrastructure. Fancourt et al.’s (2026) “arts exposome” concept is relevant here: arts-based interventions should not be conceptualized in isolation from participants’ broader patterns of daily arts engagement. The active inference framework suggests that the cumulative pattern of arts exposure, including frequency, diversity of art forms, degree of attentional engagement, and balance between active and receptive participation, shapes the maintenance and calibration of generative model parameters over time, and that intervention effects should be understood in the context of this broader exposomic pattern.

The developmental predictions elaborated in Section 12, particularly the maturation-sequence argument linking specific art forms to specific precision parameters at different developmental stages, carry substantial implications for educational policy: if confirmed, arts education would be justifiable not only on cultural grounds but as a population-level investment in allostatic health.

For cultural policy more broadly, the framework provides a mechanism-based rationale for arts investment. If the proposed pathway is empirically confirmed, then access to arts experiences that support active, agentic engagement would constitute a modifiable determinant of health, and inequities in cultural access would contribute to health inequities. Social prescribing programs connecting patients with community arts activities (Drinkwater et al., 2019; Anfilogoff et al., 2017) would gain a theoretical foundation that specifies what these programs should contain and how their effectiveness should be measured (allostatic load biomarkers, interoceptive accuracy, heart rate variability). These policy implications are conditional on empirical confirmation and should be pursued with appropriate caution.

## 15 Conclusion

This review has proposed a neurobiological framework linking arts engagement to health outcomes through the convergence of active inference and allostasis. The framework identifies a specific computational pathway: arts engagement provides settings that exercise hierarchical predictive processing, whose chronic failure produces allostatic load, and provides a schematic computational specification identifying the generative model structure, the relevant precision parameters, and the expected free energy decomposition. Arts engagement is positioned not as the exclusive route to allostatic recalibration but as a distinctively broad-spectrum form of agentic experience, one that engages multiple hierarchical levels simultaneously, prioritizes epistemic over pragmatic value, and sustains productive uncertainty in ways that purely goal-directed activities cannot match. A systematic comparison with six alternative frameworks (relaxation response, social bonding, emotional regulation, broaden-and-build, polyvagal theory, and the 50-mechanism taxonomy of Fancourt et al., 2026) shows that the active inference account can integrate the contributions of each within a common computational architecture while generating predictions that none alone can produce. Developmental and lifespan perspectives identify critical-period effects, adolescent sensitivity, and aging-related maintenance as specific predictions with educational and public health implications.

This framework is offered as the opening of an empirical research program, not as an established reference. Its value will be determined by whether its specific predictions such as non-monotonic metabolomic trajectories, ordered sensitivity of allostatic load components, partially distinguishable art-form-specific biomarker signatures, three-network convergence in neuroimaging, critical-period effects on allostatic calibration, survive systematic testing. If they do, the implications are substantial: the health effects of aesthetic experience would be characterized not as pleasant side effects of a cultural activity but as direct consequences of exercising the anticipatory, model-based processes on which physiological regulation depends. The arts, in this framework, address the organism’s regulatory architecture at its core: the capacity to predict well, to act effectively, and to maintain the dynamic equilibrium on which adaptive function rests. If this is the case, the arts may prove to be not a pleasant luxury but a pillar of human adaptive function.
