# Correction: Arts engagement, active inference, and allostatic regulation: a neurobiological framework for understanding the health effects of aesthetic experience

**DOI:** 10.3389/fncom.2026.2004139

**Published:** 2026-10-13

**Authors:** Pier Luigi Sacco

**Affiliations:** 1 Department of Neuroscience, Imaging and Clinical Sciences, University “G. d'Annunzio” of Chieti-Pescara, Chieti, Italy; 2 metaLAB (at) Harvard, Cambridge, MA, United States

**Keywords:** active inference, allostasis, allostatic load, arts and health, interoception, metabolomics, neuroaesthetics, predictive processing

In the published article, the equation in section **6. Toward a computational specification: a generative model for arts-mediated allostatic recalibration**, paragraph 5, was erroneously given as:

$$\begin{array}{l} G\left(\pi \right)\approx -{𝔼}_{\text{Q}}\left[lnP\left(o|C\right)\right]+{𝔼}_{\text{Q}}\left[D_{\text{KL}}\left[Q\left(s|o,\pi \right)\|Q\left(s|\pi \right)\right]\right] \end{array}$$

The correct equation is:

$$\begin{array}{l} G\left(\pi \right)\approx -{𝔼}_{\text{Q}}\left[lnP\left(o|C\right)\right]-{𝔼}_{\text{Q}}\left[D_{\text{KL}}\left[Q\left(s|o,\pi \right)\|Q\left(s|\pi \right)\right]\right] \end{array}$$

In the published article, in section **6. Toward a computational specification: a generative model for arts-mediated allostatic recalibration**, paragraph 5, the two terms of the decomposition were described without indicating that both enter the expression with a negative sign, leaving the relation between the minimization of expected free energy and the maximization of pragmatic and epistemic value unstated.

A correction has been made to the sentence:

“where the first term is the pragmatic value (expected log-likelihood of preferred observations *o* given prior preferences *C*) and the second is the epistemic value (the expected information gain about hidden states *s* from future observations).”

The corrected sentence reads:

“where the first term is the (negative) pragmatic value (the expected log probability of observations *o* under the prior preferences *C*) and the second is the (negative) epistemic value (the expected information gain about hidden states *s* from future observations), so that the policies with the lowest expected free energy are those with the greatest combined pragmatic and epistemic value.”

In the published article, the findings of Salimpoor et al. (2011) were erroneously reported without the anatomical dissociation established in that study between the anticipation of peak emotional responses to music and their experience. A correction has been made to section **2. Predictive processing and active inference**, paragraph 4. The sentence previously read:

“The specific relevance of this mechanism to aesthetic experience is suggested by Salimpoor et al.'s (2011) demonstration that dopamine is released in the ventral striatum during anticipation of peak emotional responses to music, though this finding is best characterized as evidence for anticipatory reward signaling rather than for epistemic value in the technical active inference sense.”

The corrected sentence reads:

“The specific relevance of this mechanism to aesthetic experience is suggested by Salimpoor et al.'s (2011) demonstration that intense musical pleasure is accompanied by striatal dopamine release, with the caudate more involved during the anticipation of peak emotional responses and the nucleus accumbens during their experience, though the anticipatory component is best characterized as evidence for anticipatory reward signaling rather than for epistemic value in the technical active inference sense.”

In the published article, Tarr et al. (2014) was erroneously described as an original demonstration, whereas the cited work is a review of the evidence on synchronized movement and endorphin release. A correction has been made to section **11. Comparison with alternative frameworks**, paragraph 1. The sentence previously read:

“Tarr et al. (2014) specifically demonstrated that synchronized movement produces elevated pain thresholds (a proxy for endorphin release) and increased feelings of social closeness and proposed that this mechanism may have co-evolved with music and dance as a means of maintaining social cohesion in groups larger than those manageable through dyadic grooming.”

The corrected sentence reads:

“Tarr et al. (2014) reviewed evidence that synchronized movement produces elevated pain thresholds (a proxy for endorphin release) and increased feelings of social closeness, and proposed that this mechanism may have co-evolved with music and dance as a means of maintaining social cohesion in groups larger than those manageable through dyadic grooming.”

The original version of this article has been updated.

